# The respiratory microbiome and susceptibility to influenza virus infection

**DOI:** 10.1371/journal.pone.0207898

**Published:** 2019-01-09

**Authors:** Kyu Han Lee, Aubree Gordon, Kerby Shedden, Guillermina Kuan, Sophia Ng, Angel Balmaseda, Betsy Foxman

**Affiliations:** 1 Department of Epidemiology, School of Public Health, University of Michigan, Ann Arbor, Michigan, United States of America; 2 Department of Biostatistics, School of Public Health, University of Michigan, Ann Arbor, Michigan, United States of America; 3 Department of Statistics, University of Michigan, Ann Arbor, Michigan, United States of America; 4 Centro de Salud Sócrates Flores Vivas, Ministry of Health, Managua, Nicaragua; 5 Sustainable Sciences Institute, Managua, Nicaragua; 6 National Virology Laboratory, Centro Nacional de Diagnóstico y Referencia, Ministry of Health, Managua, Nicaragua; University of Toronto, CANADA

## Abstract

Influenza is a major cause of morbidity and mortality worldwide. However, vaccine effectiveness has been low to moderate in recent years and vaccine coverage remains low, especially in low- and middle-income countries. Supplementary methods of prevention should be explored to reduce the high burden of influenza. A potential target is the respiratory tract microbiome, complex microbial communities which envelop the respiratory epithelium and play an important role in shaping host immunity. Using a household transmission study, we examined whether the nose/throat microbiota was associated with influenza susceptibility among participants exposed to influenza virus in the household. Further, we characterized changes in the nose/throat microbiota to explore whether community stability was influenced by influenza virus infection. Using a generalized linear mixed effects model, we found a nasal/oropharyngeal community state type (CST) associated with decreased susceptibility to influenza. The CST was rare and transitory among young children but a prevalent and stable CST among adults. Using boosting and linear mixed effects models, we found associations between the nose/throat microbiota and influenza also existed at the taxa level, specifically with the relative abundance of *Alloprevotella*, *Prevotella*, and *Bacteroides* oligotypes. We found high rates of change between bacterial community states among both secondary cases and household contacts who were not infected during follow up. Further work is needed to separate the effect of influenza virus infection from the considerable short-term changes that occur even in the absence of virus. Lastly, age was strongly associated with susceptibility to influenza and the nose/throat bacterial community structure. Although additional studies are needed to determine causality, our results suggest the nose/throat microbiome may be a potential target for reducing the burden of influenza.

## Introduction

Influenza is a major contributor of human illness and death worldwide, estimated to cause 3–5 million cases of severe illness [1] and 400,000 deaths during interpandemic years [2]. Vaccination is the best available means of influenza prevention. However, vaccine effectiveness has been low to moderate in recent years [3,4] and vaccine coverage remains low, especially in low- and middle-income countries [5]. With increasing support for a role of the microbiome in shaping host immunity [6–8], exploring whether these effects extend to influenza risk could contribute to supplementary methods of prevention.

We hypothesized that the nose/throat microbiome is an unrecognized factor associated with susceptibility to influenza virus. Murine and human studies support this assertion. Compared to controls, mice treated with oral antibiotics exhibited enhanced degeneration of the bronchiole epithelial layer and increased risk of death following intranasal infection with influenza virus [7]. In two separate randomized controlled trials, newborns fed prebiotics and probiotics had significantly lower incidence of respiratory tract infections compared to placebo [9,10]. These studies suggest the manipulation of the microbiome, either through disruption or supplementation, can alter risk of respiratory tract infections.

The epithelial cells of the upper and lower respiratory tracts are the primary targets for influenza virus infection and replication [11]. However, these cells are enveloped by complex bacterial communities that may directly or indirectly interact with influenza virus to mediate risk of infection. Commensal bacteria may prevent infection by regulating innate and adaptive host immune responses [6,7]. In addition, this immune response might stimulate changes in the microbiome [12–14]. In a human experimental trial, young adults given intranasal administration of live attenuated influenza vaccine were characterized by increased taxa richness relative to the control group [15].

Further, influenza-related changes in the bacterial community structure might explain the enhanced risk of bacterial pneumonia and otitis media following influenza virus infection [16–19]. The most commonly detected causative organisms of bacterial pneumonia and otitis media increase in abundance in the upper respiratory tract following respiratory virus infection [20,21]. We previously showed that adults in the US with influenza virus infection expressed increased nose/throat carriage of *Streptococcus pneumoniae* and *Staphylococcus aureus* [20]. Similarly, other studies have observed an increase in pneumococcal density following rhinovirus infection [21] and changes in the microbiota during rhinovirus and respiratory syncytial virus infections [22]. Increased carriage elevates risk of invasive disease [23,24], potentially through more frequent microaspiration into the lung or migration to the middle ear [25]. However, an association between the nose/throat microbiome and influenza risk has not been demonstrated in human populations.

**Fig 1:**
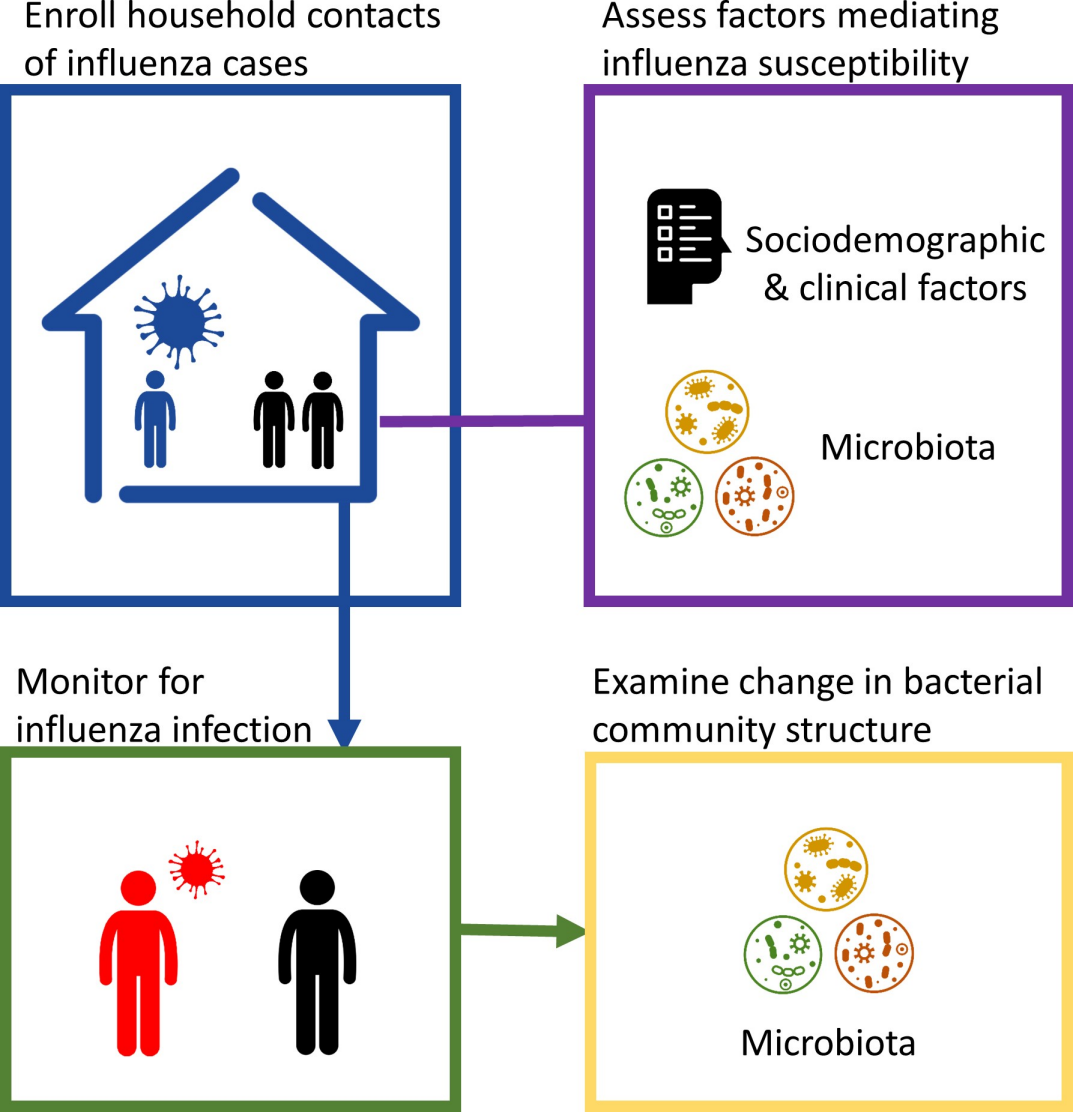
Graphical abstract.

In this study, we used data from a longitudinal household transmission study of influenza to assess the relationship between the nose/throat microbiota and susceptibility to influenza virus infection and to determine whether influenza virus infection alters the bacterial community structure using an untargeted 16S rRNA taxonomic screen (Fig 1).

## Results

### Study population

A total of 717 participants from 144 households were enrolled in the Nicaraguan Household Transmission Study during 2012–2014. During this period, 3,101 pooled nose/throat samples were collected over 5 home visits (mean: 4.3 samples per person; interquartile range (IQR): 4–5). Analysis was restricted to 537 household contacts who were negative for influenza virus by real-time reverse transcription polymerase chain reaction (RT-PCR) at time of enrollment.

Sixty-one household contacts were children ≤5 years of age (median: 2 years; IQR: 1–4), 163 were children 6–17 years of age (median: 10 years; IQR: 8–14), and 313 were adults (median: 33 years; IQR: 24–43) (Table 1). Fifty-one percent of all household contacts were exposed to at least one tobacco smoker in the household and 29% resided in crowded households (on average, >3 persons per bedroom). Household contacts were rarely vaccinated against influenza (5%) and very few used antibiotics (<1% two weeks prior to enrollment and <1% during follow up) or oseltamivir (6% during follow up).

**Table 1:** Characteristics of 537 household contacts of influenza cases from 144 households, Managua, Nicaragua, 2012–2014, by nasal/oropharyngeal community state type (CST)* at enrollment.

Characteristic	All (n = 537^†^)	CST 1 (n = 132)	CST 2 (n = 125)	CST 3 (n = 122)	CST 4 (n = 85)	CST 5 (n = 59)
	No. (%)	No. (%)	No. (%)	No. (%)	No. (%)	No. (%)
Age (years)						
0–5	61 (11)	9 (7)	15 (12)	4 (3)	3 (4)	26 (44)
6–17	163 (30)	51 (39)	41 (33)	38 (31)	19 (22)	9 (15)
≥18	313 (58)	72 (55)	69 (55)	80 (66)	63 (74)	24 (41)
Female	347 (65)	86 (65)	88 (70)	87 (71)	45 (53)	35 (59)
Influenza infection	71 (13)	21 (16)	20 (16)	15 (12)	5 (6)	6 (10)
Influenza vaccination^‡^	27 (5)	8 (6)	6 (5)	8 (7)	2 (3)	3 (5)
Smoker in household	245 (51)	54 (46)	58 (52)	56 (51)	44 (59)	28 (50)
>3 persons per bedroom in the household	156 (29)	40 (30)	35 (28)	36 (30)	22 (26)	19 (32)
Antibiotic use <2 weeks prior	1 (0)	0 (0)	0 (0)	0 (0)	0 (0)	1 (2)
Antibiotic use during follow up	4 (1)	2 (2)	0 (0)	1 (1)	0 (0)	1 (2)
Oseltamivir use during follow up	33 (6)	4 (3)	14 (11)	6 (5)	3 (4)	5 (8)

*Defined using Dirichlet multinomial mixture method (see Methods).

^†^Includes 10 household contacts with undefined CST at time of enrollment

^‡^Prior to enrollment and in same year as index case

After the enrollment of an index case, households were followed for up to 13 days through 5 home visits conducted at 2–3 day intervals. Seventy-one secondary cases from 48 households were identified by RT-PCR during follow up. Fourteen out of the 48 households had more than one secondary case (29%), suggesting clustering of cases by household. Most secondary cases were older children and young adults (median: 13.0 years; IQR: 6, 23) and had at least one symptom of an acute respiratory infection during follow up (79%) (S1 Table).

### Nasal/oropharyngeal community state types

We conducted 16S (V4) rRNA sequencing on a pair of samples from each study participant: 712 samples collected at enrollment and 698 samples collected at the last available home visit. The median time between samples was 9 days (IQR: 9–10 days). After quality filtering, microbiota data was available for 710 samples collected at enrollment and 695 samples collected at the last available home visit.

**Fig 2:**
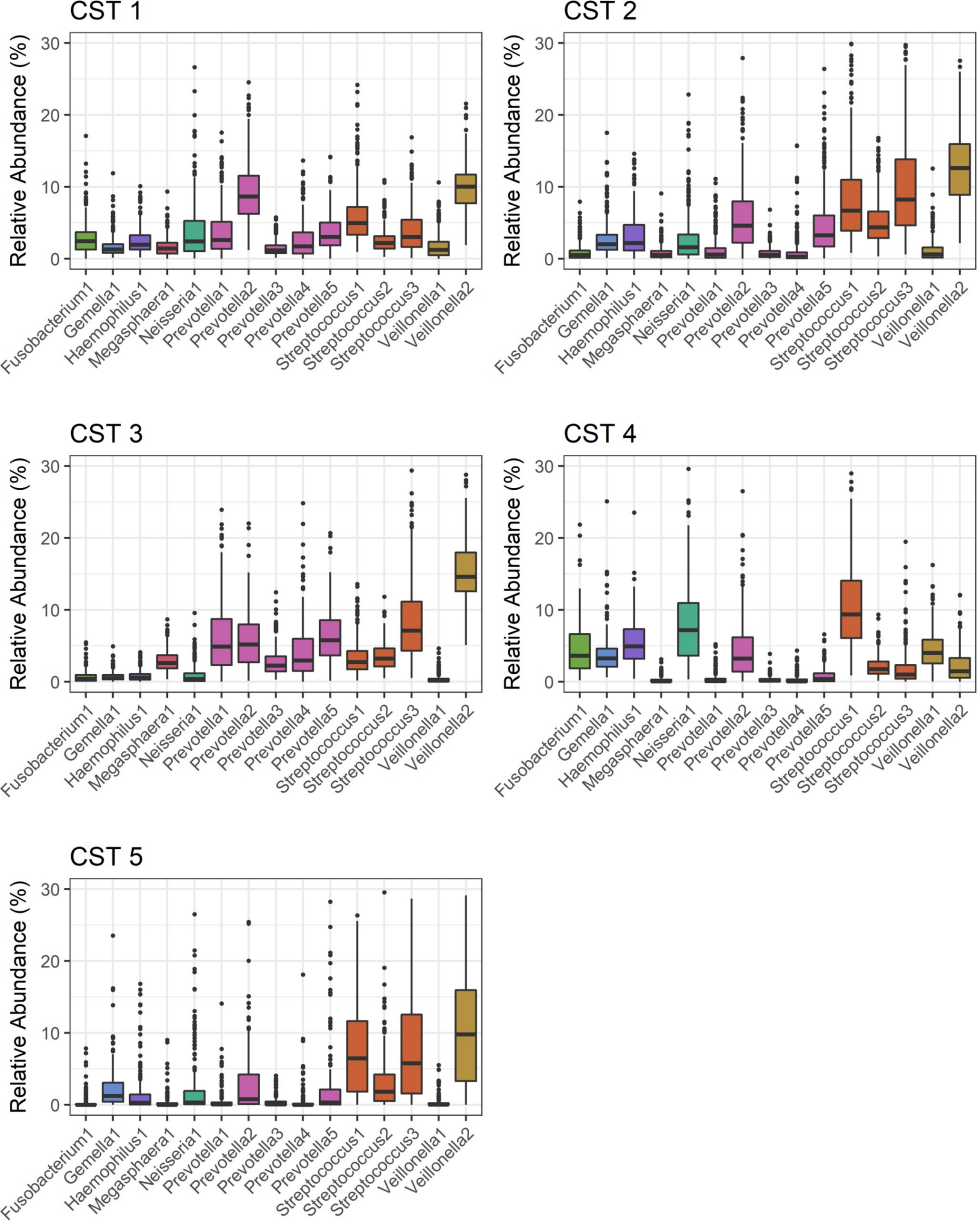
Relative abundance of 15 oligotypes, by nasal/oropharyngeal community state type (CST). Oligotypes included in the figure attributed >50% of the difference between the single-CST model and the five-CST model. Bars represent the mean relative abundance of each oligotype (±1 standard error). 1,405 samples from 717 study participants residing in 144 households in Managua, Nicaragua, 2012–2014.

Dirichlet multinomial mixture modeling [26], an unsupervised clustering method, was used to assign nose/throat samples to 5 nasal/oropharyngeal (NOP) community state types (CST) (S1 Fig: model fit by Dirichlet components; S2 Fig: PCoA plot by CST). Ninety-eight percent of all sequenced samples were assigned to a CST, after applying a ≥80% posterior probability threshold. Permutational multivariate analysis of variance (PERMANOVA) indicated NOP CSTs differed significantly from one another (Bray-Curtis dissimilarity, p = 0.001, R^2^ = 0.21). Relatively few oligotypes explained clustering of the single-CST model for the five-CST model, as 50% of the difference between models was attributed to 15 of the 230 total oligotypes. The relative abundances of these 15 oligotypes are depicted in Fig 2. The relative abundances of all 230 oligotypes are available in S2 Table.

The prevalence of NOP CSTs among household contacts differed significantly by age. Most notably, NOP CST 4 was rare among young children and became more prevalent with age (at enrollment; 0–5 years: 5%, 6–17 year: 12%, adults: 20%; χ2-test, p = 0.004) (Table 1). We observed similar results after restricting our analysis to household contacts who remained influenza negative during follow up (at enrollment; 0–5 years: 7%, 6–17 years: 12%, adults: 21%; χ2 test, p = 0.011) (S3 Table). Young children were primarily colonized by NOP CST 5, which was less common among older age groups (at enrollment 0–5 years: 43%, 6–17 years: 6%, adults: 8%; χ2-test, p<0.001) (Table 1). These results indicate age is strongly associated with the nose/throat bacterial community structure.

### Nasal/oropharyngeal community state type associated with lower susceptibility to influenza virus infection

**Fig 3:**
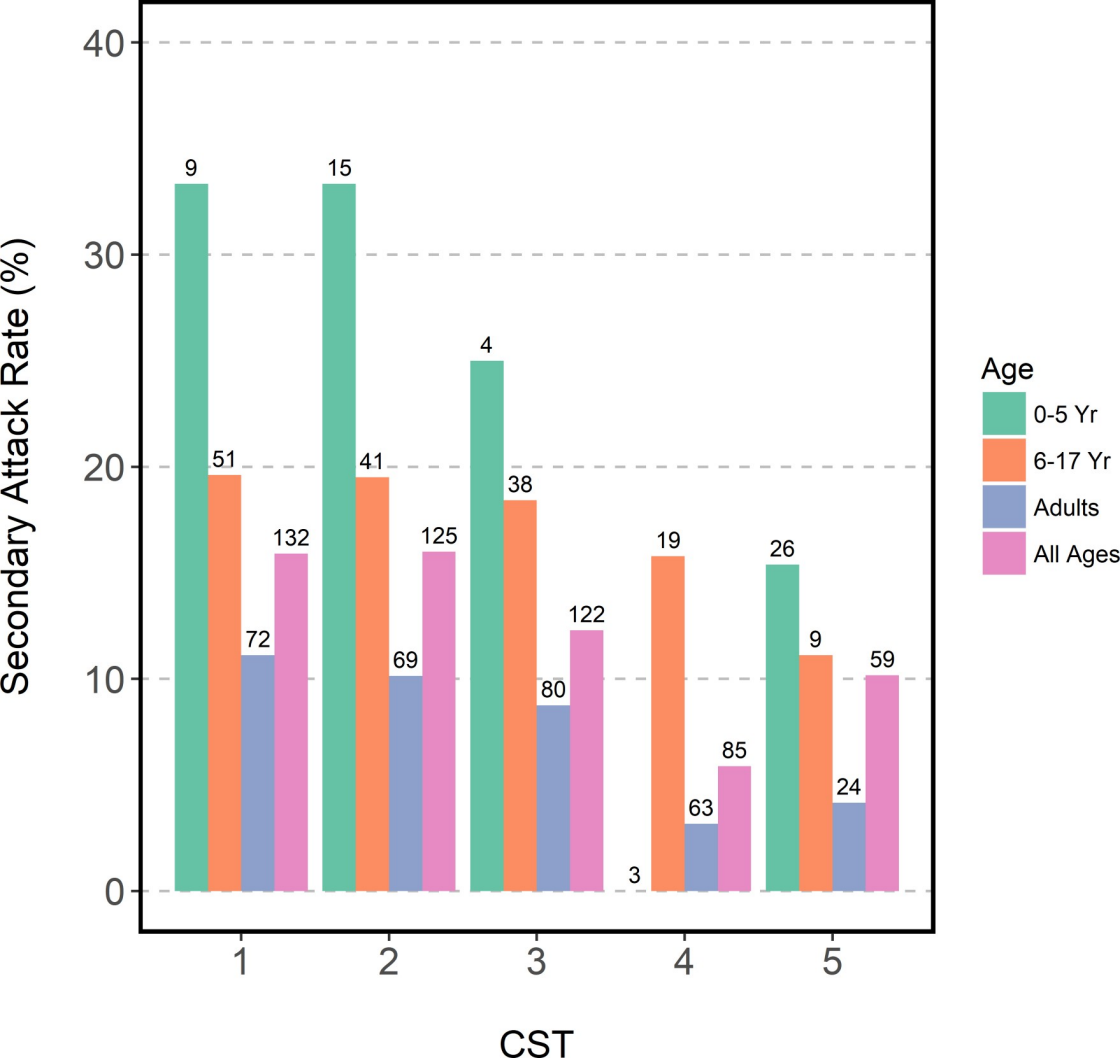
Secondary attack rates by nasal/oropharyngeal community state type at enrollment and age. 533 household contacts of influenza cases with defined community state type at enrollment, residing in 144 households in Managua, Nicaragua, 2012–2014. Numbers represent sample size of each group.

To investigate the relationship between NOP CSTs and influenza susceptibility, we first estimated secondary attack rates by NOP CST. Secondary attack rates were calculated as: the number of secondary cases identified by RT-PCR during follow up over the total number of household contacts at start of follow up. Point estimates of secondary attack rates among household contacts with NOP CST 4 were nearly half of other NOP CSTs; however, differences were not statistically significant (5.9% vs. 10.2%-16.0%; χ^2^-test, p = 0.056) (Fig 3). Similar patterns in point estimates were observed after stratifying by age. An attack rate of 0 for NOP CST 4 among young children was likely attributable to the low numbers of children with NOP CST 4 (n = 3).

**Fig 4:**
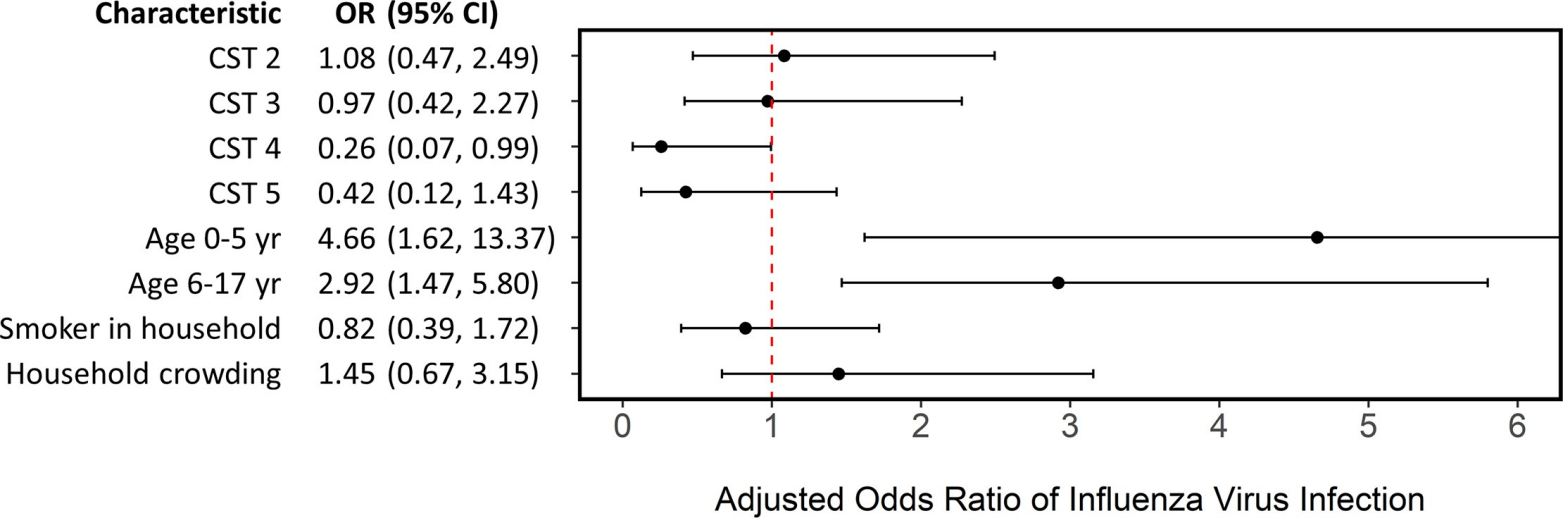
Generalized linear mixed effects model estimating odds of influenza virus infection. Model adjusting for nasal/oropharyngeal community state type (relative to community state type 1), age (relative to adults), a smoker in the household, household crowding (average of >3 persons per bedroom), and clustering by household. 468 household contacts of influenza cases with complete data, residing in 132 households in Managua, Nicaragua, 2012–2014.

We used a generalized linear mixed effects model to examine the relationship between NOP CSTs and influenza susceptibility after adjusting for age, a smoker in the household, household crowding, and clustering by household. A detailed description of the model is available in S1 Appendix. Our decision to account for household clustering was supported by an intra-class correlation of 0.21, which indicates 21% of the total variance was due to clustering by household. We found household contacts with NOP CST 4 had a lower odds of influenza virus infection (odds ratio (OR): 0.26; 95% CI: 0.07, 0.99) (Fig 4), Further, young children were most likely to acquire influenza virus (OR: 4.66; 95% CI: 1.62, 13.37), followed by older children (OR: 2.91; 95% CI: 1.47, 5.80). These results suggest household contacts with NOP CST 4 were less likely to be infected with influenza and younger household contacts were at greater risk after adjusting for other known risk factors.

We were inadequately powered for influenza type/subtype-specific models; however, no household contacts with NOP CST 4 at enrollment (n = 85) were infected with H3N2, the most commonly identified influenza subtype in this population (52% of all secondary cases). This suggests associations between the microbiota and influenza susceptibility may vary by subtype but further work is needed to test this hypothesis.

### Oligotypes associated with susceptibility to influenza virus infection

In addition to analysis at the CST level, taxa-specific analysis was conducted using MaAsLin [27]. MaAsLin first uses boosting in a univariate pre-screen to identify taxa and metadata (features) that are potentially associated. Significantly associated features are then identified using linear mixed effects models. Models included in our analysis adjusted for age, a smoker in the household, household crowding, and clustering by household (S1 Appendix). Two oligotypes, *Alloprevotella sp*. and *Prevotella histicola / sp*. */ veroralis / fusca / scopos / melaninogenica*, were positively associated with influenza virus infection (S4 Table). One oligotype was negatively associated with influenza virus infection. Although unclassified in the HOMD database, a BLAST search using the GenBank database (https://www.ncbi.nlm.nih.gov/genbank/) classified the oligotype as *Bacteroides vulgatus*. All three oligotypes were rare in all NOP CSTs (S2 Table) and in the total community composition (range: 0.01–0.38%).

The relative abundance of multiple oligotypes were strongly associated with age. Relative to adults, 119 oligotypes were differentially abundant among young children and 41 oligotypes were differentially abundant among older children. Lastly, four oligotypes were associated with household crowding and no oligotype was associated with exposure to a smoker in the household. All statistically significant associations are listed in S4 Table.

### Community diversity and influenza virus infection

We examined whether community diversity was associated with influenza susceptibility. Shannon diversity was significantly different between NOP CST 4 and other NOP CSTs (Wilcox rank-sum tests, all comparisons p<0.001) (S3 Fig). NOP CST 4 (median: 3.43) was less diverse than NOP CST 1 (median: 3.58) and more diverse than NOP CSTs 2, 3, and 5 (medians: 2.56–3.29).

To further explore whether community diversity influenced the relationship between NOP CSTs and influenza susceptibility, we reran our generalized linear mixed effects model using Shannon diversity as our primary predictor. Alpha diversity was not significantly associated with influenza susceptibility (OR: 1.76; 95% CI: 0.83, 3.72) (S4 Fig).

### Stability of community state types during influenza virus infection

**Fig 5:**
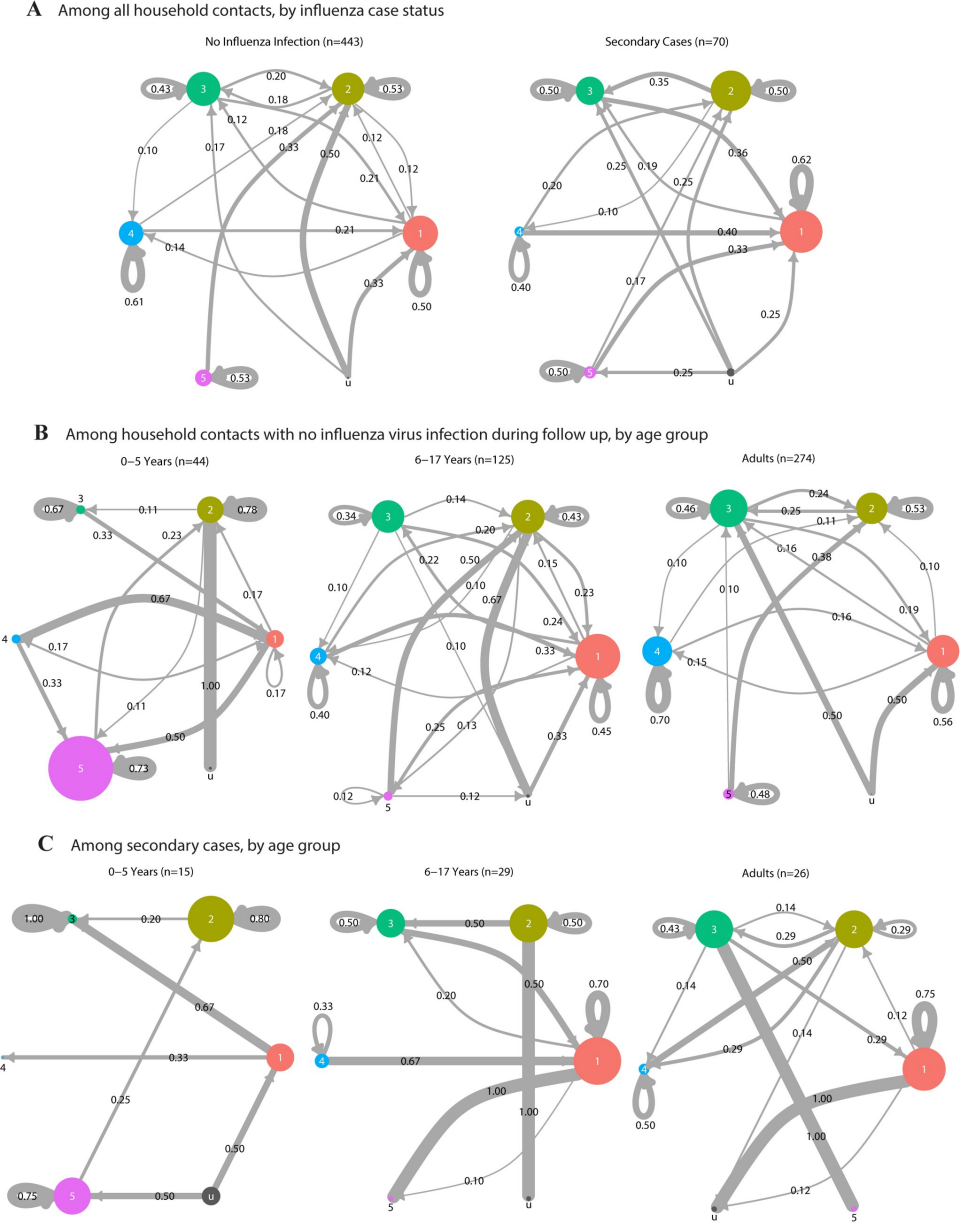
Stability of nasal/oropharyngeal community state type (CST) over follow up. 513 household contacts with microbiota data both at enrollment and follow up, residing in 144 households in Managua, Nicaragua, 2012–2014. **(A)** By influenza case status. **(B)** By age, among 443 household contacts who remained negative for influenza virus infection during follow up. **(C)** By age, among 70 secondary cases. Circles represent nasal/oropharyngeal community state types (CST) and circle size is proportional to prevalence of CSTs at enrollment. CST u corresponds to samples with an undefined CST. Transition rates between CSTs were estimated as Markov chain probabilities and are shown numerically. Transitions rates <0.10 were removed for simplicity. Complete data are available in S2 Appendix.

To examine the stability of the respiratory microbiota during influenza virus infection, we characterized changes in the bacterial community structure over a median of 9 days (IQR: 9, 10). We used Markov chain plots to represent short-term changes in the nose/throat microbiota among household contacts, by influenza case status and age (Fig 5 and S2 Appendix). Circles represent NOP CST 1 through 5 and the size of the circles represent the prevalence of each CST at start of follow up. Arrows represent transitions between CSTs during follow up. The width and number assigned to each arrow represents the proportion of individuals who transitioned between CSTs. CST stability was estimated as the proportion of household contacts who showed no change in CST over follow up.

Although the prevalence of NOP CSTs appeared to remain similar between the two sampling points (S3 Table), we found transitions between CSTs were common with approximately half of all household contacts (45% among secondary cases, 55% among uninfected) changing to a different CST by the end of follow up (Fig 5 and S2 Appendix). Stability ranged from 40–62% for all CSTs in both secondary cases and uninfected household contacts. Although we were inadequately powered to test for statistical differences in specific CST-to-CST transitions, the overall contrast between the two groups suggest community dynamics may differ by influenza status and should be explored further.

We specifically focused on the stability of NOP CST 4, which was associated with decreased influenza susceptibility. Stability among uninfected household contacts with NOP CST 4 increased with age (0–5 years: 0%, 6–17 years: 40%, adults: 70%; Fisher exact test, p = 0.016) (Fig 5B). We were inadequately powered for a similar analysis among secondary cases.

**Fig 6:**
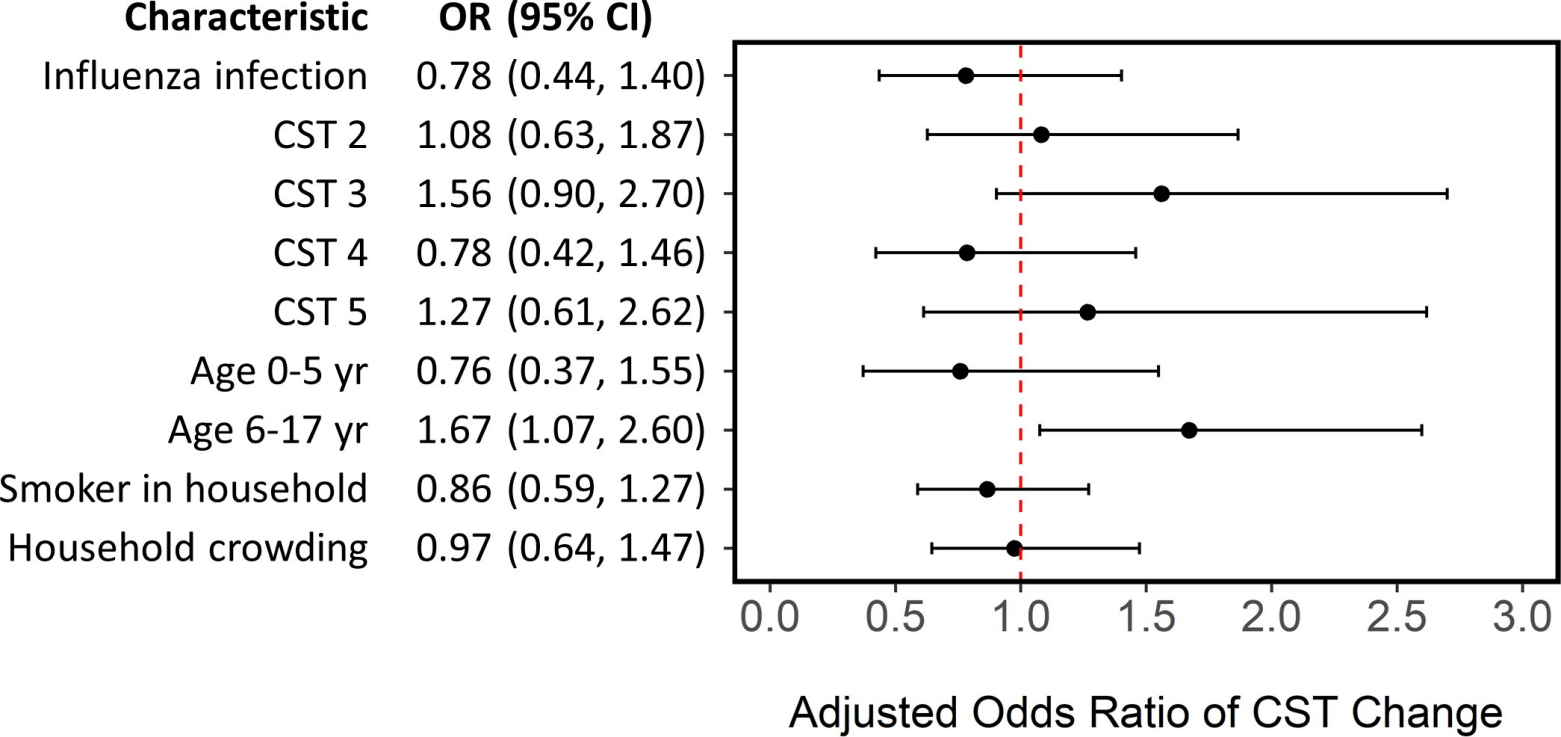
Generalized linear mixed effects model estimating odds of change in nasal/oropharyngeal community state type (CST) during follow up. Model adjusting for influenza virus infection, nasal/oropharyngeal community state type at enrollment (relative to CST 1), age (relative to adults), a smoker in the household, household crowding (average of >3 persons per bedroom), and clustering by household. 443 household contacts with defined CST at enrollment and follow up and complete data, residing in 130 households in Managua, Nicaragua, 2012–2014. Household contacts with an undefined CST were excluded from analysis.

We used a generalized linear mixed effects model to examine whether NOP CST stability was associated with influenza virus infection, after adjusting for NOP CST at enrollment, age, a smoker in the household, household crowding, and clustering by household (S1 Appendix). We did not find an association between NOP CST stability and influenza virus infection. However, we found stability was lowest among children 6–17 years old (OR: 1.67; 95% CI: 1.07, 2.60) (Fig 6).

### Sensitivity analysis

Sensitivity analysis was conducted to investigate potential sources of bias (S3 Appendix). To assess whether sequencing depth could influence our results, we first examined whether sequencing depth differed by NOP CST. We found no meaningful differences in sequencing depth by NOP CST. In addition, we reran our influenza susceptibility model with sequencing depth as an additional predictor. We found only minor differences in model estimates, with a slightly enhanced effect of NOP CST 4 (OR: 0.24; 95% CI: 0.06, 0.94).

To assess whether time between samples influenced CST stability, we additionally controlled for time between samples in our CST stability model. We found only minor differences in model estimates.

We explored whether a more conservative criterion for NOP CST assignments would influence our results. We reran our influenza susceptibility model after assigning samples with a maximum posterior probability <90% as missing. We found minor differences in our results. However, the association with NOP CST 4 was no longer statistically significant (OR: 0.27; 95% CI: 0.07, 1.03).

Lastly, we examined whether the microbiome was associated with prior infection and immunity. We used generalized linear mixed effects models to examine whether a hemagglutinin inhibition (HAI) titer of ≥1:40 was associated with NOP CST, after adjusting for age and clustering by household. A titer of 1:40 is commonly associated with a 40–70% reduced risk of influenza [28]. We ran this model for each influenza subtype and repeated the analysis using a more flexible titer of ≥1:20. We found no statistically significant association between titer and NOP CST.

## Discussion

To our knowledge, this is the first human population study to prospectively explore the relationship between the nose/throat microbiome and influenza virus infection. Although secondary attack rates did not differ by NOP CST, we demonstrate influenza susceptibility is associated with differences in the overall bacterial community structure after adjusting for potential confounders. The exact biological mechanisms remain unclear but the few murine studies that have examined this relationship suggest it is likely mediated by immunomodulation. In these studies, mice treated with antibiotics exhibited diminished innate and adaptive immune responses compared to placebo. Specifically, mice with disrupted microbiomes expressed impaired macrophage responses to type I and type II interferons and lacked microbiomes containing bacterial lipopolysaccharides that stimulate Toll-like receptors and other pattern recognition receptors [6,7]. Although these mechanisms suggest a causal relationship between the respiratory microbiome and influenza virus infection, additional work is needed to evaluate whether epidemiologic associations in human populations represent a true effect of the microbiome or merely reflect differences in host immunity. Further, future studies should examine whether the relationship differs by influenza type/subtype.

Most longitudinal studies that have characterized the upper respiratory tract microbiota are limited to infants [22,29,30]. Here, we examine a unique population consisting of both children and adults. We demonstrate age is strongly associated with both the prevalence and stability of nose/throat bacterial communities. Most notably, we found the NOP CST associated with decreased susceptibility to influenza was less prevalent and less stable among young children. If a causal relationship between the microbiome and influenza truly exists, our results would suggest the microbiome may contribute to the increased influenza risk observed among young children [31].

We found the microbiome structure changed frequently among both influenza cases and household contacts who remained uninfected during follow up (median: 9.0 days, IQR: 9.0–10.0). This was expected among influenza cases as prior studies have demonstrated increased colonization by opportunistic pathogens in the upper respiratory tract following respiratory virus infection [20,21]. However, much less is known for short-term changes in the upper respiratory tract microbiome of healthy individuals, especially among adults. The high degree of change among uninfected household contacts in our study may represent normal variation among healthy individuals or be indicative of a response to influenza exposure in the household.

Preliminary findings from our Markov chain analysis suggest community dynamics may differ by influenza status. Characterizing multiple longitudinal samples per participant would lead to a better understand of influenza and its impact on the microbiome. In addition, future studies should explore whether changes in the bacterial community structure are directly due to influenza virus or indirect responses to changes in the virome or mycobiome.

A limitation in our study is the use of pooled nose and throat samples. Differential sampling by site can introduce bias if sampling is related to both the observed bacterial community structure and influenza susceptibility. Although this was minimized through consistent sampling techniques across all study participants, factors such as age could confound associations and may partially explain age-related differences in the bacterial community structure. In our analysis, we used descriptive statistics to thoroughly explore the effects of age and other potential confounders on our outcomes and adjusted as appropriate in our statistical models. A second limitation is the use of RT-PCR for identifying influenza cases. Individuals can be infected with influenza virus (i.e. ≥4-fold increase in hemagglutinin inhibition antibody titer) and not shed virus [32]. We may have missed true index cases and secondary cases with low levels of virus. Although our results and conclusions are limited to secondary cases with viral shedding, RT-PCR allowed us to screen for secondary cases at 2–3 day intervals while limiting invasive procedures. Lastly, we did not consider pre-existing immunity from previous infections in our models, which might potentially confound or modify associations. However, we did not find any statistically significant associations between HAI titer and NOP CSTs in our sensitivity analysis.

While much work is needed to translate these results into potential clinical and public health applications, our findings contribute to a growing literature suggesting that it may be possible to manipulate the microbiome and decrease risk of disease [9,10]. Influenza virus is a major cause of severe illness and death each year [1,2]. However, vaccine effectiveness varies by year [4] and there still much debate on the use of antivirals for prophylaxis, especially for preventing asymptomatic infections and influenza transmission [33]. Our study suggests the microbiome should be further explored as a potential target in reducing influenza risk.

## Methods

### Study population and sample collection

The Nicaraguan Household Transmission Study of Influenza is an ongoing prospective case-ascertained study conducted among urban households in Managua, Nicaragua. Patients attending the Health Center Sócrates Flores Vivas were screened for study eligibility. Index cases of influenza were identified as patients with a positive QuickVue Influenza A+B rapid test, symptom onset of an acute respiratory infection within the past 48 hours, and living with at least one other household member. Symptoms of acute respiratory infection included fever or feverishness with cough, sore throat, or runny nose.

Index cases and household members (contacts) were invited to participate and clinical, sociodemographic, and household data were collected at time of enrollment. Participants were followed for up to 13 days through 5 home visits conducted at 2–3 day intervals. At each home visit, oropharyngeal and anterior nares swabs were collected, combined, and stored at 4°C in viral transport media. All samples were transported to the National Virology Laboratory at the Nicaraguan Ministry of Health within 48 hours of collection and stored at -80°C. A symptom diary was collected for all participants during follow up.

A total of 168 households were enrolled for follow up during 2012–2014. Households were excluded from analysis if a suspected index case was negative for influenza virus by real-time reverse-transcription polymerase chain reaction (RT-PCR) at time of enrollment. RT-PCR was used to identify influenza cases as infections are often asymptomatic [34]. Two household contacts were excluded from analysis due to missing influenza virus infection status at time of enrollment. The remaining participants consisted of 144 index cases of influenza positive by RT-PCR, 537 household contacts influenza negative by RT-PCR at time of enrollment, and 36 household contacts who were RT-PCR positive for influenza virus on the first day of follow up.

### Ethics statement

Written informed consent was obtained from adult participants and from parents or legal guardians of participants under 18 years of age. In addition, verbal assent was obtained from children over 5 years of age. The study was approved by Institutional Review Boards at the University of Michigan, the Nicaraguan Ministry of Health, and the University of California at Berkeley.

### RNA extraction and RT-PCR

Total RNA was extracted from all available nasal/oropharyngeal samples using the QIAmp Viral Mini Kit (QIAGEN, Hilden, Germany) per manufacturer’s instructions at the National Virology Laboratory in Nicaragua. Samples were tested for influenza virus by RT-PCR using standard protocols validated by the Centers for Disease Control and Prevention [35].

### DNA extraction and 16S rRNA sequencing

Total DNA was extracted from a pair of samples from each study participant: the first sample collected at time of enrollment and the second sample collected at the last day of follow up (median days between samples: 9.0 days, IQR: 9.0–10.0). Among the 717 total study participants, five first samples and 19 second samples were not available for DNA extraction. DNA was extracted using the QIAmp DNA Mini Kit and an enzyme cocktail composed of cell lysis solution (Promega, Madison, USA), lysozyme, mutanolysin, RNase A, and lysostaphin (Sigma-Aldrich, St. Lious, USA) in 22.5:4.5:1.125:1.125:1 parts, respectively. 100 μL of sample was incubated at 37°C for 30 minutes with 80 μL of the enzyme cocktail. After adding 25 μL proteinase K and 200 μL of Buffer AL, samples were vortexed and incubated at 56°C for 30 minutes. Samples were washed with 200 μL of 100% ethanol, 500 uL of Buffer AW1, and then 500 uL of Buffer AW2. To maximize DNA yield, DNA was eluted twice with 100 uL of Buffer AE and stored at -80°C.

The V4 hypervariable region of the 16S rRNA gene was amplified and sequenced at the University of Michigan Microbial Systems Laboratories using Illumina MiSeq V2 chemistry 2x250 (Illumina, San Diego, CA) and a validated dual-indexing method [36]. Briefly, primers consisted of an Illumina adapter, an 8-nt index sequence, a 10-nt pad sequence, a 2-nt linker, and the V4-specific F515/R806 primer [37]. Amplicons were purified and pooled in equimolar concentrations. A mock community of 21 species (Catalog No. HM-782D, BEI Resources, Manassas, VA) or a mock community of 10 species (Catalog No. D6300, Zymo Research, Irvine, CA) was included by the Microbial Systems Laboratories to assess sequencing error rates. For every 96-well plate submitted for amplification and sequencing (90 study samples), we included two aliquots of an in-house mock community consisting of *Streptococcus pneumoniae*, *Streptococcus pyogenes*, *Staphylococcus aureus*, *Haemophilus influenzae*, and *Moraxella catarrhalis* and two aliquots of an oropharyngeal control sample. These internal controls were randomly assigned to plate wells and used to assess systematic variation in sequencing. All samples were sequenced in duplicate, demultiplexed, and quality filtered.

### Oligotyping and community state types

We used mothur v1.38.1 [38] to align and perform quality filtering on raw sequences using the mothur standard operating procedures (https://www.mothur.org/wiki/MiSeq_SOP, accessed November 18, 2016). Sequences were converted to the appropriate oligotyping format as previously described [39]. We used the Minimum Entropy Decomposition (MED) algorithm [40] with default parameters (-M: 13779.0, -V: 3 nt) to cluster sequences into oligotypes. Briefly, the algorithm identifies variable nucleotide positions and uses Shannon entropy to partition sequences into nodes. The process is iterative and continues to decompose parent nodes into child nodes until there are no discernable entropy peaks. Oligotyping has previously been used to examine within-genus variations in the microbiota [39,41–43] and provides increased resolution relative to conventional distance-based clustering methods.

After excluding five samples with less than 1,000 reads, our dataset consisted of 1,405 samples with a total of 61,784,957 sequences decomposed into 230 oligotypes. To assign taxonomy, we searched representative sequences of each oligotype against the Human Oral Microbiome Database (HOMD) v14.51 [44] using blastn v2.2.23 [45]. Classifications with ≥98% identity were kept.

We used Dirichlet multinomial mixture models [26] in R v3.4.4 [46] and the DirichletMultinomial v1.16.0 package [47] to assign all samples to 5 NOP CSTs. We determined the number of CSTs by comparing the Laplace approximation of the negative log models and identifying the point at which an increase in Dirichlet components resulted in minor reductions in model fit (S1 Fig). This approach allowed us to consider both model fit of the negative log models and statistical power in downstream analysis. The goal was not to identify the “true communities”, as CSTs are representations of data. All formal statistical inferences are based on the models relating CSTs to our outcomes of interest, with any findings being statistically supported by the data.

Samples were assigned to NOP CSTs with the greatest posterior probability. 98.2% of all samples had a posterior probability of 80% or higher. To minimize misclassification, samples were assigned as having an undefined NOP CST if the posterior probability was less than 80%. Each NOP CST contained between 13.0–24.8% of all samples (n = 182–348) and 1.8% of all samples (n = 25) were undefined.

### Statistical models

Detail to statistical models used in this study are described in S1 Appendix. To examine the association between NOP CSTs at enrollment and susceptibility of influenza virus infection, we used a generalized linear mixed effects model estimating the odds of infection after adjusting for NOP CST (relative to NOP CST 1), age (relative to adults), a smoker in the household, household crowding, and clustering by household. Household crowding was defined as having, on average, more than three household members per bedroom. The model was adapted to examine the effects of alpha diversity.

Associations between individual oligotypes and participant data were determined using MaAsLin [27]. Briefly, MaAsLin is a sparse multivariate approach used to identify associations between individual taxa and participant data. Relative abundance values were arcsine square-root transformed to stabilize variance. Potentially associated features (i.e. oligotypes) were selected using boosting in a univariate prescreen. Linear mixed effects models are then used to find associations between the selected features and metadata. The Benjamin-Hochberg method was used to correct for multiple testing. Associations with a q-value <0.05 were considered statistically significant.

To examine the effect of influenza virus infection on community stability, we used a generalized linear mixed effects model estimating the odds of any change in NOP CST over follow up, after adjusting for NOP CST at enrollment (relative to NOP CST 1), age (relative to adults), a smoker in the household, household crowding (average of >3 persons per bedroom), and clustering by household. All statistical analysis was conducted using R v3.4.4 [46] and the lme4, vegan, and maaslin packages [27,48,49].

### Markov chain analysis

We estimated CST transition rates over time using methods described previously [50]. Briefly, we restricted our dataset to household contacts with complete nose/throat sample pairs (i.e. microbiota data at enrollment and at follow up). CST transition rates were calculated as Markov chain probabilities. Analysis was repeated after stratifying by influenza status and age.

## Sensitivity analysis

We conducted sensitivity analyses to investigate potential sources of bias including sequencing depth, follow up time, NOP CST assignment, and prior infection and immunity (S3 Appendix). To assess sequencing depth, we examined the distribution of total sequences per sample by CST. We then reran our influenza susceptibility model after additionally adjusting for sequencing depth. To examine whether time between sampling points could influence our results, we reran our community stability model after additionally adjusting for time between samples. We also examined whether using a more conservative criterion for CST assignments could affect our results by rerunning our influenza susceptibility model and community stability model after assigning samples to missing if the maximum posterior probability was less than 90%. Lastly, we examined whether HAI titer was associated with NOP CSTs. For each influenza subtype, we ran generalized linear mixed effects models that examined the relationship between a titer ≥1:40 and NOP CSTs, after adjusting for age and clustering by household. Models were repeated using a more flexible titer cutoff of ≥1:20.

## Supporting information

S1 Table Characteristics of 71 secondary cases from 48 households, Managua, Nicaragua, 2012–2014, by nasal/oropharyngeal community state type (CST) at enrollment. (DOCX)

S2 Table Relative abundance of all 230 oligotypes, by nasal/oropharyngeal community state type (CST). (XLSX)

S3 Table Distribution of nasal/oropharyngeal community state types by age, time, and whether acquired influenza by end of follow up. (DOCX)

S4 Table MaAsLin results. (XLSX)

S1 Fig Model fit of negative log models by number of Dirichlet components. We determined the number of nasal/oropharyngeal community state types (CST) by estimating the Laplace approximation of the negative log models and identifying the point at which an increase in Dirichlet components resulted in minor reductions in model fit. This approach allowed us to consider both model fit of the negative log models and statistical power in downstream analysis. The goal was not to identify the “true communities”, as CSTs are representations of data. All formal statistical inferences are based on the models relating CSTs to our outcomes of interest, with any findings being statistically supported by the data.
(TIF)

S2 Fig Principal coordinates analysis of nose/throat samples assigned to nasal/oropharyngeal community state types. 1,405 nose/throat samples from 717 study participants residing in 144 households in Managua, Nicaragua, 2012–2014. Based on Bray-Curtis dissimilarity.
(TIF)

S3 Fig Shannon diversity of nose/throat samples, by nasal/oropharyngeal community state type. 1,380 samples with defined community state types, 717 study participants residing in 144 households in Managua, Nicaragua, 2012–2014.
(TIF)

S4 Fig Generalized linear mixed effects model estimating odds of influenza virus infection using Shannon diversity. Model adjusts for Shannon diversity, age (relative to adults), a smoker in the household, household crowding (average of >3 persons per bedroom), and clustering by household. 477 household contacts of influenza cases with complete data, residing in 132 households in Managua, Nicaragua, 2012–2014.
(TIF)

S1 Appendix Description of statistical models. (DOCX)

S2 Appendix Stability of nose/throat community state type (CST) over follow up, including all transitions. (DOCX)

S3 Appendix Sensitivity analysis. (DOCX)
