# Supplementary material for: The respiratory microbiome and susceptibility to influenza virus infection
Source: PLoS One. 2019 Jan 9;14(1):e0207898. doi: 10.1371/journal.pone.0207898 (PMC6326417; doi:10.1371/journal.pone.0207898)
Supplement: S1 Table — (DOCX) [file pone.0207898.s001.docx]

**S1 Table. Characteristics of 71 secondary cases from 48 households, Managua, Nicaragua, 2012-2014, by community type at enrollment.**

| **Characteristic** | **All**  **(n=71^†^)** | **Community Type 1**  **(n=21)** | **Community Type 2**  **(n=20)** | **Community Type 3**  **(n=15)** | **Community Type 4**  **(n=5)** | **Community Type 5**  **(n=6)** |
| --- | --- | --- | --- | --- | --- | --- |
|  | **No. (%)** | **No. (%)** | **No. (%)** | **No. (%)** | **No. (%)** | **No. (%)** |
| Age (years) |  |  |  |  |  |  |
| 0-5 | 15 (21) | 3 (14) | 5 (25) | 1 (7) | 0 (0) | 4 (67) |
| 6-17 | 30 (42) | 10 (48) | 8 (40) | 7 (47) | 3 (60) | 1 (17) |
| ≥18 | 26 (37) | 8 (38) | 7 (35) | 7 (47) | 2 (40) | 1 (17) |
| Female | 43 (61) | 12 (57) | 15 (75) | 9 (60) | 3 (60) | 3 (50) |
| Influenza vaccination^‡^ | 2 (3) | 0 (0) | 1 (5) | 1 (8) | 0 (0) | 0 (0) |
| Smoker in household | 29 (52) | 7 (39) | 9 (53) | 6 (43) | 3 (100) | 3 (50) |
| >3 persons per bedroom in the household | 25 (35) | 8 (38) | 8 (40) | 4 (27) | 2 (40) | 1 (17) |
| Antibiotic use  <2 weeks prior | 0 (0) | 0 (0) | 0 (0) | 0 (0) | 0 (0) | 0 (0) |
| Antibiotic use during follow up | 1 (1) | 0 (0) | 0 (0) | 1 (7) | 0 (0) | 0 (0) |
| Oseltamivir use during follow up | 9 (13) | 2 (10) | 4 (20) | 2 (13) | 0 (0) | 1 (17) |
| ARI^§^ symptom | 56 (79) | 15 (71) | 17 (85) | 13 (87) | 5 (100) | 4 (67) |
| Fever | 37 (52) | 10 (48) | 12 (60) | 6 (40) | 4 (80) | 3 (50) |
| Cough | 43 (61) | 9 (43) | 16 (80) | 11 (73) | 4 (80) | 3 (50) |
| Sore throat | 29 (41) | 8 (38) | 11 (55) | 7 (47) | 1 (20) | 2 (33) |
| Runny nose | 42 (59) | 10 (48) | 15 (75) | 10 (67) | 3 (60) | 3 (50) |

^†^Includes secondary cases with undefined community types

^‡^Prior to enrollment and in same year as index case

^§^Acute respiratory infection
