# Supplementary material for: The respiratory microbiome and susceptibility to influenza virus infection
Source: PLoS One. 2019 Jan 9;14(1):e0207898. doi: 10.1371/journal.pone.0207898 (PMC6326417; doi:10.1371/journal.pone.0207898)
Supplement: S3 Table — (DOCX) [file pone.0207898.s003.docx]

**S3 Table.** **Distribution of community types by age, time, and whether acquired influenza by end of follow up.** 537 household contacts with any microbiota data, residing in 144 households in Managua, Nicaragua, 2012-2014.

|  | **Secondary Case**  **(71 Household Contacts)** | | | | **No Influenza Infection**  **(466 Household Contacts)** | | | |
| --- | --- | --- | --- | --- | --- | --- | --- | --- |
|  | **All** | **0-5 Yr** | **6-17 Yr** | **Adults** | **All** | **0-5 Yr** | **6-17 Yr** | **Adults** |
|  | **No. (%)** | **No. (%)** | **No. (%)** | **No. (%)** | **No. (%)** | **No. (%)** | **No. (%)** | **No. (%)** |
| All Samples | n=141 | n=30 | n=59 | n=52 | n=909 | n=90 | n=258 | n=561 |
| CT 1 | 44 (31) | 4 (13) | 23 (39) | 17 (33) | 222 (24) | 11 (12) | 82 (32) | 129 (23) |
| CT 2 | 35 (25) | 10 (33) | 13 (22) | 12 (23) | 221 (24) | 25 (28) | 65 (25) | 131 (23) |
| CT 3 | 34 (24) | 5 (17) | 16 (27) | 13 (25) | 196 (22) | 6 (7) | 53 (21) | 137 (24) |
| CT 4 | 11 (8) | 1 (3) | 4 (7) | 6 (12) | 158 (17) | 4 (4) | 33 (13) | 121 (22) |
| CT 5 | 11 (8) | 8 (27) | 2 (3) | 1 (2) | 99 (11) | 43 (48) | 17 (7) | 39 (7) |
| Undefined | 6 (4) | 2 (7) | 1 (2) | 3 (6) | 13 (1) | 1 (1) | 8 (3) | 4 (1) |
| At Enrollment | n=71 | n=15 | n=30 | n=26 | n=462 | n=45 | n=132 | n=285 |
| CT 1 | 21 (30) | 3 (20) | 10 (33) | 8 (31) | 111 (24) | 6 (13) | 41 (31) | 64 (22) |
| CT 2 | 20 (28) | 5 (33) | 8 (27) | 7 (27) | 105 (23) | 10 (22) | 33 (25) | 62 (22) |
| CT 3 | 15 (21) | 1 (7) | 7 (23) | 7 (27) | 107 (23) | 3 (7) | 31 (23) | 73 (26) |
| CT 4 | 5 (7) | 0 (0) | 3 (10) | 2 (8) | 80 (17) | 3 (7) | 16 (12) | 61 (21) |
| CT 5 | 6 (8) | 4 (27) | 1 (3) | 1 (4) | 53 (11) | 22 (49) | 8 (6) | 23 (8) |
| Undefined | 4 (6) | 2 (13) | 1 (3) | 1 (4) | 6 (1) | 1 (2) | 3 (3) | 2 (1) |
| At Follow Up | n=70 | n=15 | n=29 | n=26 | n=447 | n=45 | n=126 | n=276 |
| CT 1 | 23 (33) | 1 (7) | 13 (45) | 9 (35) | 111 (25) | 5 (11) | 41 (33) | 65 (24) |
| CT 2 | 15 (21) | 5 (33) | 5 (17) | 5 (19) | 116 (26) | 15 (33) | 32 (25) | 69 (25) |
| CT 3 | 19 (27) | 4 (27) | 9 (31) | 6 (23) | 89 (20) | 3 (7) | 22 (17) | 64 (23) |
| CT 4 | 6 (9) | 1 (7) | 1 (3) | 4 (15) | 78 (17) | 1 (2) | 17 (13) | 60 (22) |
| CT 5 | 5 (7) | 4 (27) | 1 (3) | 0 (0) | 46 (10) | 21 (47) | 9 (7) | 16 (6) |
| Undefined | 2 (3) | 0 (0) | 0 (0) | 2 (8) | 7 (2) | 0 (0) | 5 (4) | 2 (1) |
