# Supplementary figures and images for: The respiratory microbiome and susceptibility to influenza virus infection
Source: PLoS One. 2019 Jan 9;14(1):e0207898. doi: 10.1371/journal.pone.0207898 (PMC6326417; doi:10.1371/journal.pone.0207898)

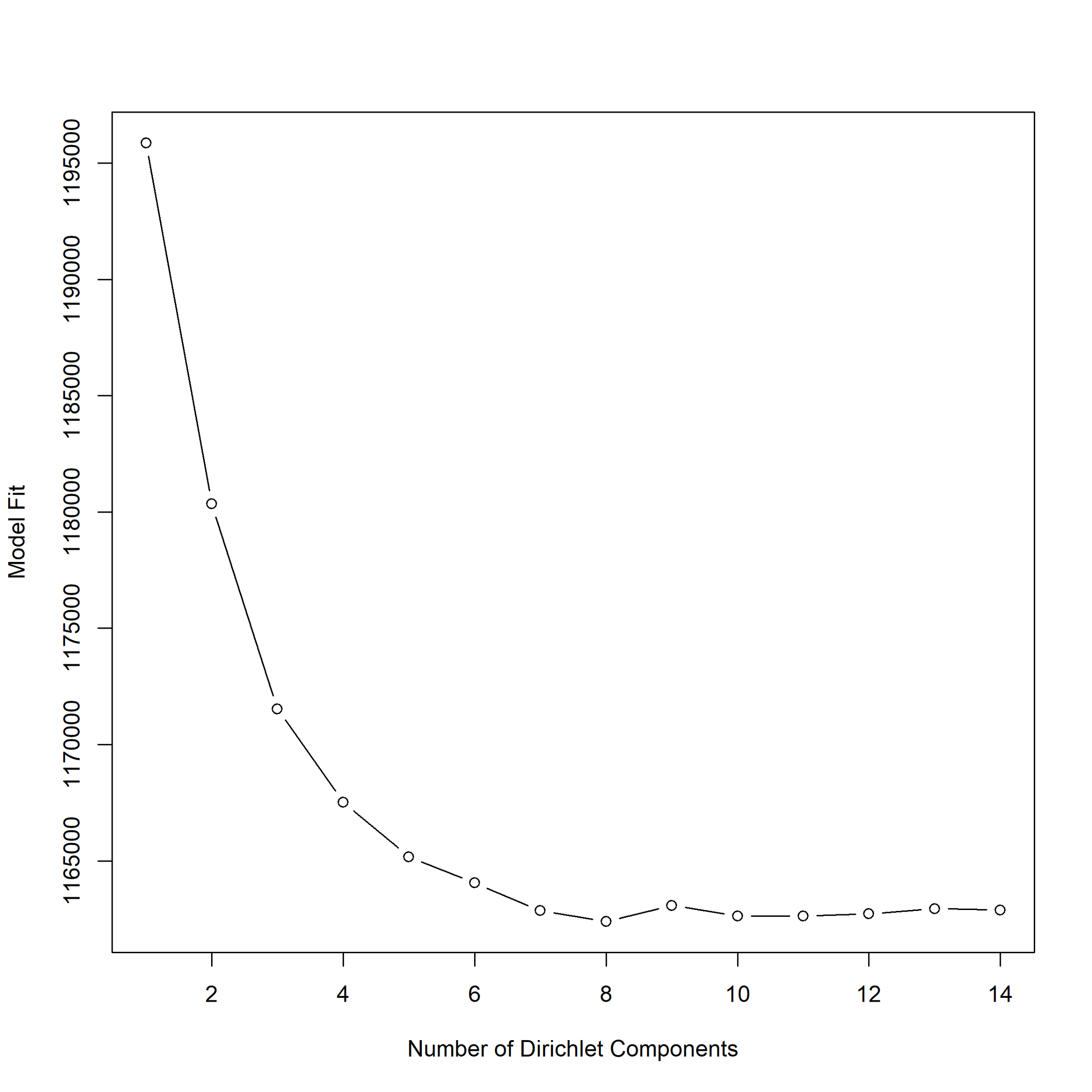

Supplement: S1 Fig — We determined the number of nasal/oropharyngeal community state types (CST) by estimating the Laplace approximation of the negative log models and identifying the point at which an increase in Dirichlet components resulted in minor reductions in model fit. This approach allowed us to consider both model fit of the negative log models and statistical power in downstream analysis. The goal was not to identify the “true communities”, as CSTs are representations of data. All formal statistical inferences are based on the models relating CSTs to our outcomes of interest, with any findings being statistically supported by the data. (TIF) [file pone.0207898.s005.tif]

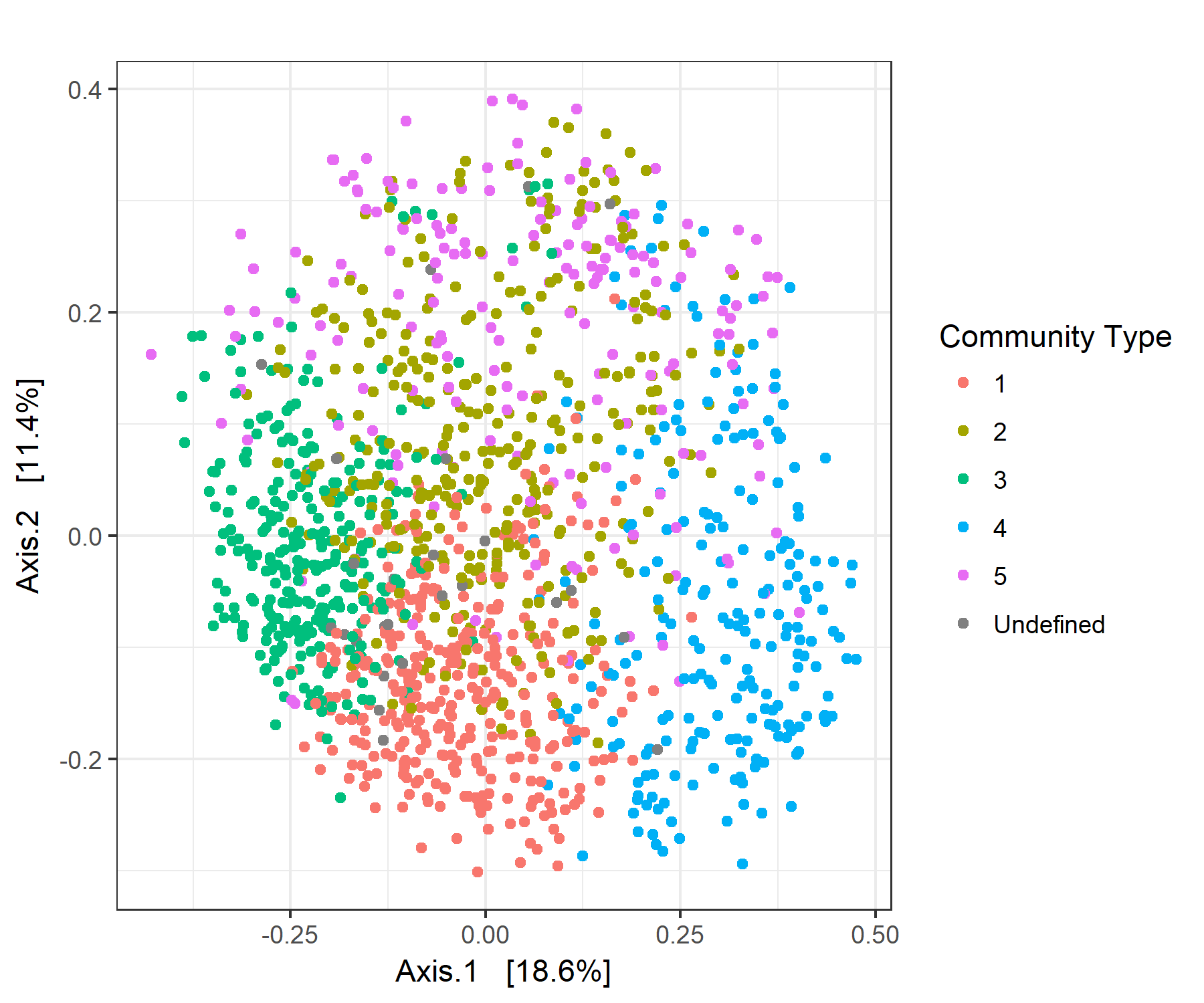

Supplement: S2 Fig — 1,405 nose/throat samples from 717 study participants residing in 144 households in Managua, Nicaragua, 2012–2014. Based on Bray-Curtis dissimilarity. (TIF) [file pone.0207898.s006.tif]

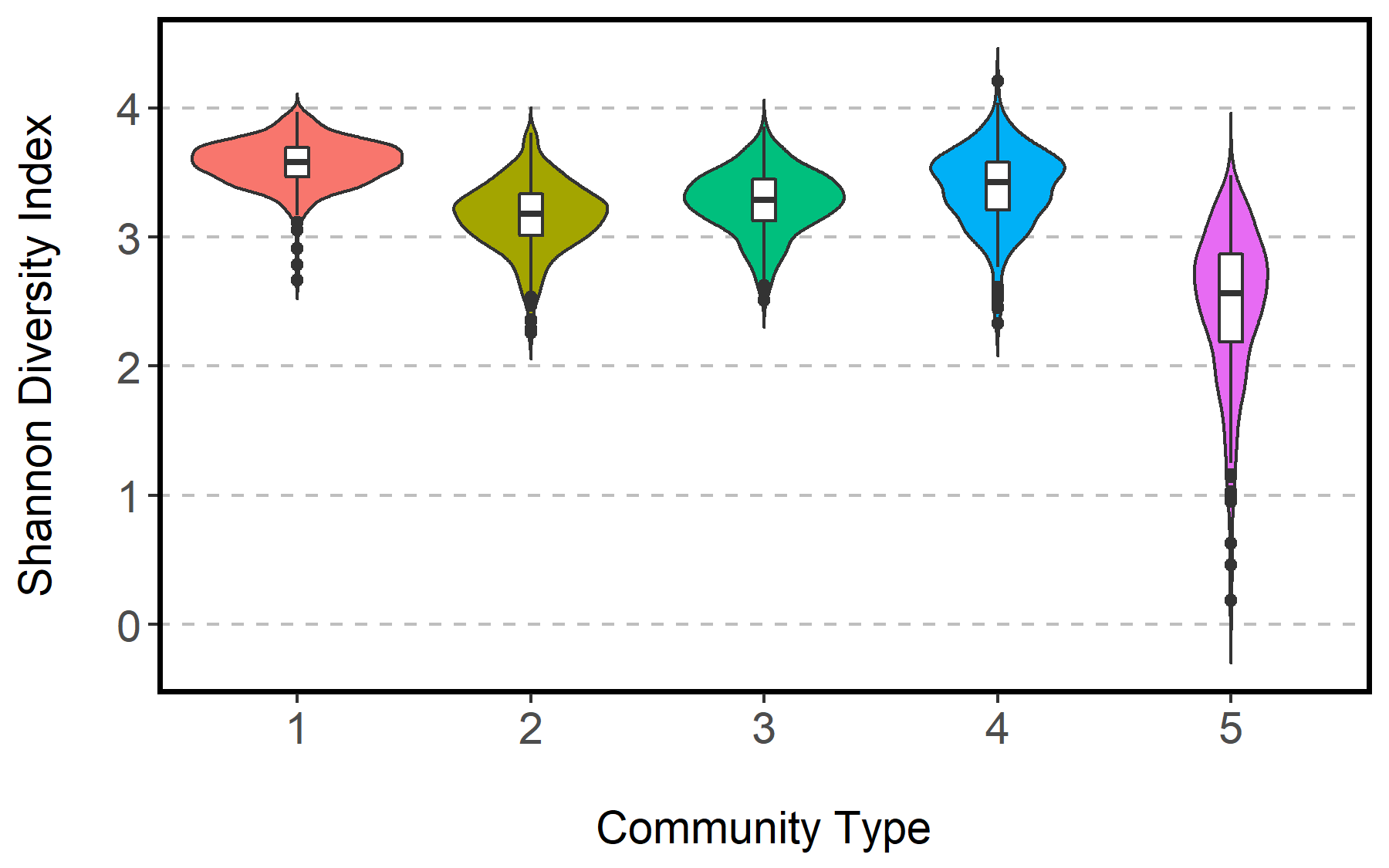

Supplement: S3 Fig — 1,380 samples with defined community state types, 717 study participants residing in 144 households in Managua, Nicaragua, 2012–2014. (TIF) [file pone.0207898.s007.tif]

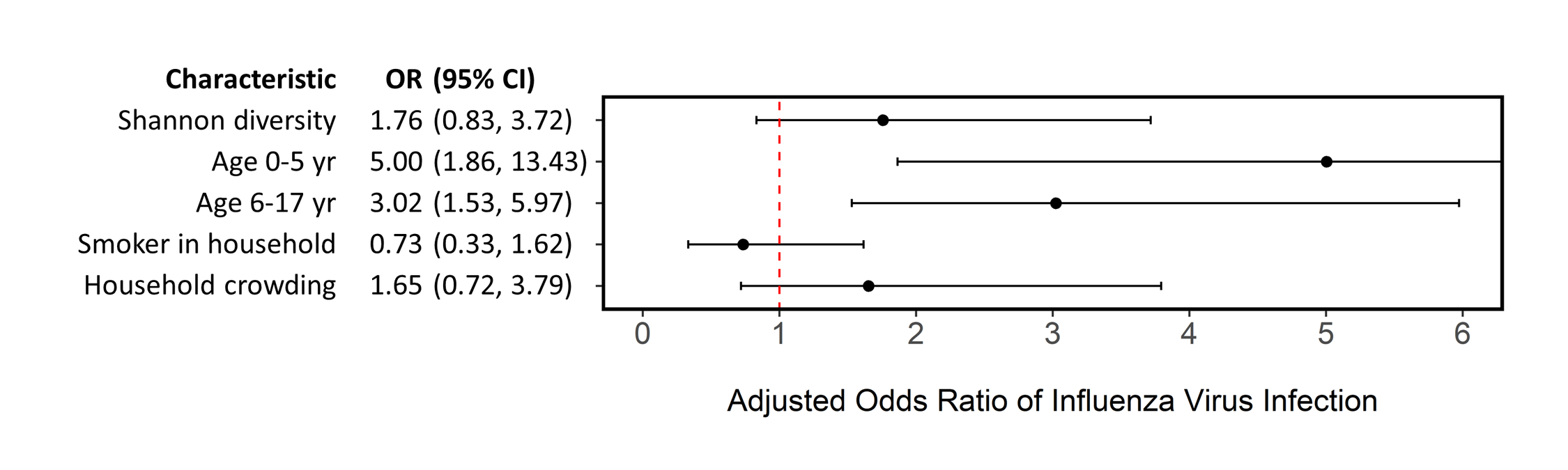

Supplement: S4 Fig — Model adjusts for Shannon diversity, age (relative to adults), a smoker in the household, household crowding (average of >3 persons per bedroom), and clustering by household. 477 household contacts of influenza cases with complete data, residing in 132 households in Managua, Nicaragua, 2012–2014. (TIF) [file pone.0207898.s008.tif]
