# Supplementary material for: The respiratory microbiome and susceptibility to influenza virus infection
Source: PLoS One. 2019 Jan 9;14(1):e0207898. doi: 10.1371/journal.pone.0207898 (PMC6326417; doi:10.1371/journal.pone.0207898)
Supplement: S1 Appendix — (DOCX) [file pone.0207898.s009.docx]

**S1 Appendix**

**Description of statistical models**

**Influenza susceptibility model using community state types (CST)**

Model:

$logit(Pr(Y_{ij}=1))= \beta_{0}+\beta_{1}{CST}_{ij}+\beta_{2}{Age}_{ij}+\beta_{3}{Smoker}_{ij}+\beta_{4}{Crowding}_{ij}+b_{0j}+\varepsilon_{ij}$

where *i* denotes person

*j* denotes household

Description:

|  | **Description** | **Variable Format** | **Type** |
| --- | --- | --- | --- |
| *Y* | Influenza virus infection during follow up | Binary factor | Outcome |
| *CST* | CST at time of enrollment | 5-level categorical factor | Fixed |
| *Age* | Age groups: 0-5 years, 6-17 years, 18-87 years | 3-level categorical factor | Fixed |
| *Smoker* | Smoker in the household | Binary factor | Fixed |
| *Crowding* | Average >3 persons per bedroom | Binary factor | Fixed |
| *b_0_* | Random effects for household | Household ID as factor | Random |
| *ε* | Random error |  | Random |

Results:

|  | **Odds Ratio** | **95% Confidence Interval** |
| --- | --- | --- |
| CST 2 | 1.08 | (0.47, 2.49) |
| CST 3 | 0.97 | (0.42, 2.27) |
| CST 4 | 0.26 | (0.07, 0.99) |
| CST 5 | 0.42 | (0.12, 1.43) |
| 0-5 years | 4.66 | (1.62, 13.37) |
| 6-17 years | 2.92 | (1.47, 5.80) |
| Smoker in household | 0.82 | (0.39, 1.72) |
| Household crowding | 1.45 | (0.67, 3.15) |

468 observations with complete data

10 degrees of freedom

**Influenza susceptibility models using MaAsLin**

Model:

$$arcsin(\sqrt{\gamma_{ij}})= \beta_{0}+\beta_{1}{Influenza}_{ij}+\beta_{2}{Age}_{ij}+\beta_{3}{Smoker}_{ij}+\beta_{4}{Crowding}_{ij}+b_{0j}+\varepsilon_{ij}$$

where *i* denotes person

*j* denotes household

Description:

|  | **Description** | **Variable Format** | **Type** |
| --- | --- | --- | --- |
| γ | Relative abundance of select oligotype | Continuous | Outcome |
| *Influenza* | Influenza status | Binary factor | Fixed |
| *Age* | Age groups: 0-5 years, 6-17 years, 18-87 years | 3-level categorical factor | Fixed |
| *Smoker* | Smoker in the household | Binary factor | Fixed |
| *Crowding* | Average >3 persons per bedroom | Binary factor | Fixed |
| *b_0_* | Random effects for household | Household ID as factor | Random |
| *ε* | Random error |  | Random |

Results available in S4 Table.

**Influenza susceptibility model using Shannon diversity**

Model:

$logit(Pr(Y_{ij}=1))= \beta_{0}+\beta_{1}{Shannon}_{ij}+\beta_{2}{Age}_{ij}+\beta_{3}{Smoker}_{ij}+\beta_{4}{Crowding}_{ij}+b_{0j}+\varepsilon_{ij}$

where *i* denotes person

*j* denotes household

Description:

|  | **Description** | **Variable Format** | **Type** |
| --- | --- | --- | --- |
| *Y* | Influenza virus infection during follow up | Binary factor | Outcome |
| *Shannon* | Shannon diversity | Continuous | Fixed |
| *Age* | Age groups: 0-5 years, 6-17 years, 18-87 years | 3-level categorical factor | Fixed |
| *Smoker* | Smoker in the household | Binary factor | Fixed |
| *Crowding* | Average >3 persons per bedroom | Binary factor | Fixed |
| *b_0_* | Random effects for household | Household ID as factor | Random |
| *ε* | Random error |  | Random |

Results:

|  | **Odds Ratio** | **95% Confidence Interval** |
| --- | --- | --- |
| Shannon diversity | 1.76 | (0.83, 3.71) |
| 0-5 years | 5.00 | (0.83, 13.43) |
| 6-17 years | 3.02 | (1.53, 5.97) |
| Smoker in household | 0.73 | (0.33, 1.62) |
| Household crowding | 1.65 | (0.72, 3.79) |

477 observations with complete data

7 degrees of freedom

**CST stability model**

Model:

$$logit(Pr(Y_{ij}=1))= \beta_{0}+\beta_{1}{CST}_{ij}+\beta_{2}{Age}_{ij}+\beta_{3}{Smoker}_{ij}+\beta_{4}{Crowding}_{ij}+b_{0j}+\varepsilon_{ij}$$

where *i* denotes person

*j* denotes household

Description:

|  | **Description** | **Variable Format** | **Type** |
| --- | --- | --- | --- |
| *Y* | Any change in CST between sampling points | Binary factor | Outcome |
| *CST* | CST at time of enrollment | 5-level categorical factor | Fixed |
| *Age* | Age groups: 0-5 years, 6-17 years, 18-87 years | 3-level categorical factor | Fixed |
| *Smoker* | Smoker in the household | Binary factor | Fixed |
| *Crowding* | Average >3 persons per bedroom | Binary factor | Fixed |
| *b_0_* | Random effects for household *j* | Household ID as categorical factor | Random |
| *ε* | Random error |  | Random |

Results:

|  | **Odds Ratio** | **95% Confidence Interval** |
| --- | --- | --- |
| Influenza virus infection | 0.78 | (0.44, 1.40) |
| CST 2 | 1.08 | (0.63, 1.87) |
| CST 3 | 1.56 | (0.90, 2.70) |
| CST 4 | 0.78 | (0.42, 1.46) |
| CST 5 | 1.27 | (0.61, 2.62) |
| 0-5 years | 0.76 | (0.37, 1.55) |
| 6-17 years | 1.67 | (1.07, 2.60) |
| Smoker in household | 0.86 | (0.59, 1.27) |
| Household crowding | 0.97 | (0.64, 1.47) |

443 observations with complete data

11 degrees of freedom
