# Supplementary material for: The respiratory microbiome and susceptibility to influenza virus infection
Source: PLoS One. 2019 Jan 9;14(1):e0207898. doi: 10.1371/journal.pone.0207898 (PMC6326417; doi:10.1371/journal.pone.0207898)
Supplement: S2 Appendix — (DOCX) [file pone.0207898.s010.docx]

**S2 Appendix**

**Stability of nose/throat community state type (CST) over follow up, including all transitions.**

513 household contacts with microbiota data at enrollment and follow up, residing in 144 households in Managua, Nicaragua, 2012-2014. Rows indicate CST at enrollment and columns indicate CST at follow up. Cells indicate transition rates over follow up for each pair of CSTs. Undefined CST indicates samples that were not assigned to any CST.

**Among all household contacts with no influenza infection (n=443)**

|  | | CST at follow up | | | | | |
| --- | --- | --- | --- | --- | --- | --- | --- |
|  |  | 1 | 2 | 3 | 4 | 5 | Undefined |
| CST at enrollment | 1 | 0.50 | 0.12 | 0.18 | 0.14 | 0.05 | 0.02 |
|  | 2 | 0.12 | 0.53 | 0.18 | 0.07 | 0.09 | 0.01 |
|  | 3 | 0.21 | 0.20 | 0.43 | 0.10 | 0.03 | 0.03 |
|  | 4 | 0.21 | 0.12 | 0.03 | 0.61 | 0.03 | 0.00 |
|  | 5 | 0.08 | 0.33 | 0.04 | 0.00 | 0.53 | 0.02 |
|  | Undefined | 0.33 | 0.50 | 0.17 | 0.00 | 0.00 | 0.00 |

**Among all secondary cases (n=70)**

|  | | CST at follow up | | | | | |
| --- | --- | --- | --- | --- | --- | --- | --- |
|  |  | 1 | 2 | 3 | 4 | 5 | Undefined |
| CST at enrollment | 1 | 0.62 | 0.05 | 0.19 | 0.05 | 0.05 | 0.05 |
|  | 2 | 0.00 | 0.50 | 0.35 | 0.10 | 0.00 | 0.05 |
|  | 3 | 0.36 | 0.07 | 0.50 | 0.07 | 0.00 | 0.00 |
|  | 4 | 0.40 | 0.20 | 0.00 | 0.40 | 0.00 | 0.00 |
|  | 5 | 0.33 | 0.17 | 0.00 | 0.00 | 0.50 | 0.00 |
|  | Undefined | 0.25 | 0.25 | 0.25 | 0.00 | 0.25 | 0.00 |

**Among household contacts with no influenza infection, 0-5 years of age (n=44)**

|  | | CST at follow up | | | | | |
| --- | --- | --- | --- | --- | --- | --- | --- |
|  |  | 1 | 2 | 3 | 4 | 5 | Undefined |
| CST at enrollment | 1 | 0.17 | 0.17 | 0.00 | 0.17 | 0.50 | 0.00 |
|  | 2 | 0.00 | 0.78 | 1.11 | 0.00 | 0.11 | 0.00 |
|  | 3 | 0.33 | 0.00 | 0.67 | 0.00 | 0.00 | 0.00 |
|  | 4 | 0.67 | 0.00 | 0.00 | 0.00 | 0.33 | 0.00 |
|  | 5 | 0.05 | 0.23 | 0.00 | 0.00 | 0.73 | 0.00 |
|  | Undefined | 0.00 | 1.00 | 0.00 | 0.00 | 0.00 | 0.00 |

**Among household contacts with no influenza infection, 6-17 years of age (n=125)**

|  | | CST at follow up | | | | | |
| --- | --- | --- | --- | --- | --- | --- | --- |
|  |  | 1 | 2 | 3 | 4 | 5 | Undefined |
| CST at enrollment | 1 | 0.45 | 0.15 | 0.23 | 0.13 | 0.05 | 0.00 |
|  | 2 | 0.23 | 0.43 | 0.07 | 0.10 | 0.13 | 0.03 |
|  | 3 | 0.24 | 0.14 | 0.34 | 0.10 | 0.07 | 0.10 |
|  | 4 | 0.33 | 0.20 | 0.07 | 0.40 | 0.00 | 0.00 |
|  | 5 | 0.25 | 0.50 | 0.00 | 0.00 | 0.13 | 0.13 |
|  | Undefined | 0.33 | 0.67 | 0.00 | 0.00 | 0.00 | 0.00 |

**Among household contacts with no influenza infection, adults (n=274)**

|  | | CST at follow up | | | | | |
| --- | --- | --- | --- | --- | --- | --- | --- |
|  |  | 1 | 2 | 3 | 4 | 5 | Undefined |
| CST at enrollment | 1 | 0.56 | 0.10 | 0.16 | 0.15 | 0.00 | 0.03 |
|  | 2 | 0.08 | 0.53 | 0.25 | 0.07 | 0.07 | 0.00 |
|  | 3 | 0.19 | 0.24 | 0.46 | 0.10 | 0.01 | 0.00 |
|  | 4 | 0.16 | 0.11 | 0.02 | 0.70 | 0.02 | 0.00 |
|  | 5 | 0.05 | 0.38 | 0.10 | 0.00 | 0.48 | 0.00 |
|  | Undefined | 0.50 | 0.00 | 0.50 | 0.00 | 0.00 | 0.00 |

**Among secondary cases, 0-5 years of age (n=15)**

|  | | CST at follow up | | | | | |
| --- | --- | --- | --- | --- | --- | --- | --- |
|  |  | 1 | 2 | 3 | 4 | 5 | Undefined |
| CST at enrollment | 1 | 0.00 | 0.00 | 0.67 | 0.33 | 0.00 | 0.00 |
|  | 2 | 0.00 | 0.80 | 0.20 | 0.00 | 0.00 | 0.00 |
|  | 3 | 0.00 | 0.00 | 1.00 | 0.00 | 0.00 | 0.00 |
|  | 4 | 0.00 | 0.00 | 0.00 | 0.00 | 0.00 | 0.00 |
|  | 5 | 0.00 | 0.25 | 0.00 | 0.00 | 0.75 | 0.00 |
|  | Undefined | 0.50 | 0.00 | 0.00 | 0.00 | 0.50 | 0.00 |

**Among secondary cases, 6-17 years of age (n=29)**

|  | | CST at follow up | | | | | |
| --- | --- | --- | --- | --- | --- | --- | --- |
|  |  | 1 | 2 | 3 | 4 | 5 | Undefined |
| CST at enrollment | 1 | 0.70 | 0.00 | 0.20 | 0.00 | 0.10 | 0.00 |
|  | 2 | 0.00 | 0.50 | 0.50 | 0.00 | 0.00 | 0.00 |
|  | 3 | 0.50 | 0.00 | 0.50 | 0.00 | 0.00 | 0.00 |
|  | 4 | 0.67 | 0.00 | 0.00 | 0.33 | 0.00 | 0.00 |
|  | 5 | 1.00 | 0.00 | 0.00 | 0.00 | 0.00 | 0.00 |
|  | Undefined | 0.00 | 1.0 | 0.00 | 0.00 | 0.00 | 0.00 |

**Among secondary cases, adults (n=26)**

|  | | CST at follow up | | | | | |
| --- | --- | --- | --- | --- | --- | --- | --- |
|  |  | 1 | 2 | 3 | 4 | 5 | Undefined |
| CST at enrollment | 1 | 0.75 | 0.13 | 0.00 | 0.00 | 0.00 | 0.13 |
|  | 2 | 0.00 | 0.29 | 0.29 | 0.29 | 0.00 | 0.14 |
|  | 3 | 0.29 | 0.14 | 0.43 | 0.14 | 0.00 | 0.00 |
|  | 4 | 0.00 | 0.50 | 0.00 | 0.50 | 0.00 | 0.00 |
|  | 5 | 1.00 | 0.00 | 0.00 | 0.00 | 0.00 | 0.00 |
|  | Undefined | 0.00 | 0.00 | 1.00 | 0.00 | 0.00 | 0.00 |
