# Supplementary material for: The respiratory microbiome and susceptibility to influenza virus infection
Source: PLoS One. 2019 Jan 9;14(1):e0207898. doi: 10.1371/journal.pone.0207898 (PMC6326417; doi:10.1371/journal.pone.0207898)
Supplement: S3 Appendix — (DOCX) [file pone.0207898.s011.docx]

**S3 Appendix**

**Sensitivity analysis**

**Sequencing depth by nasal/oropharyngeal community state type (CST).** 1,380 samples with defined CST from 717 study participants residing in 144 households in Managua, Nicaragua, 2012-2014.


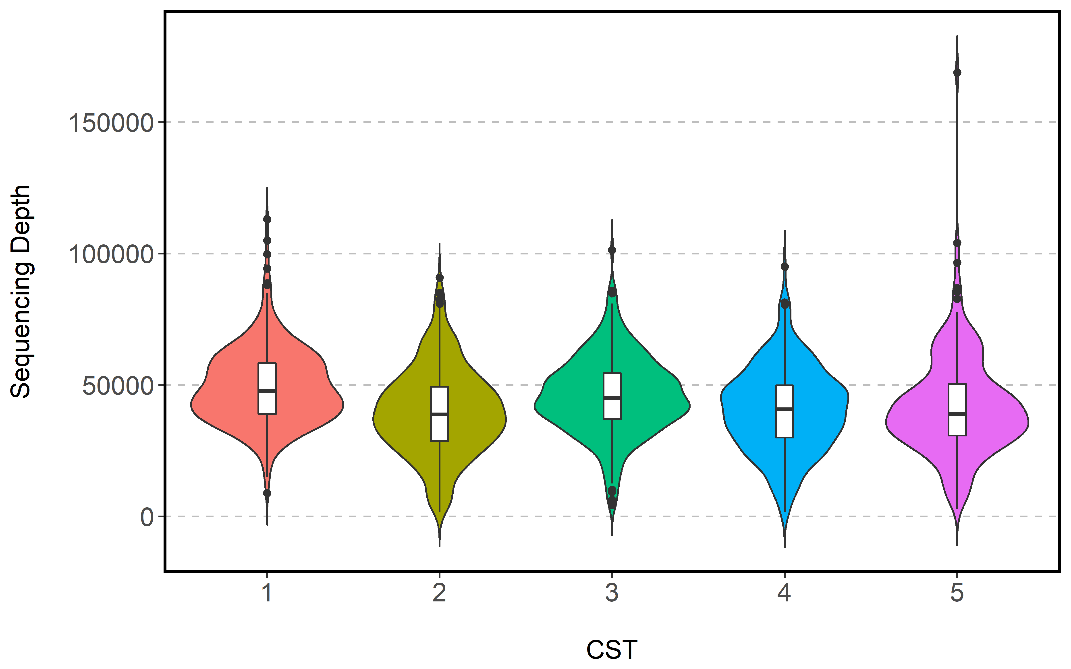


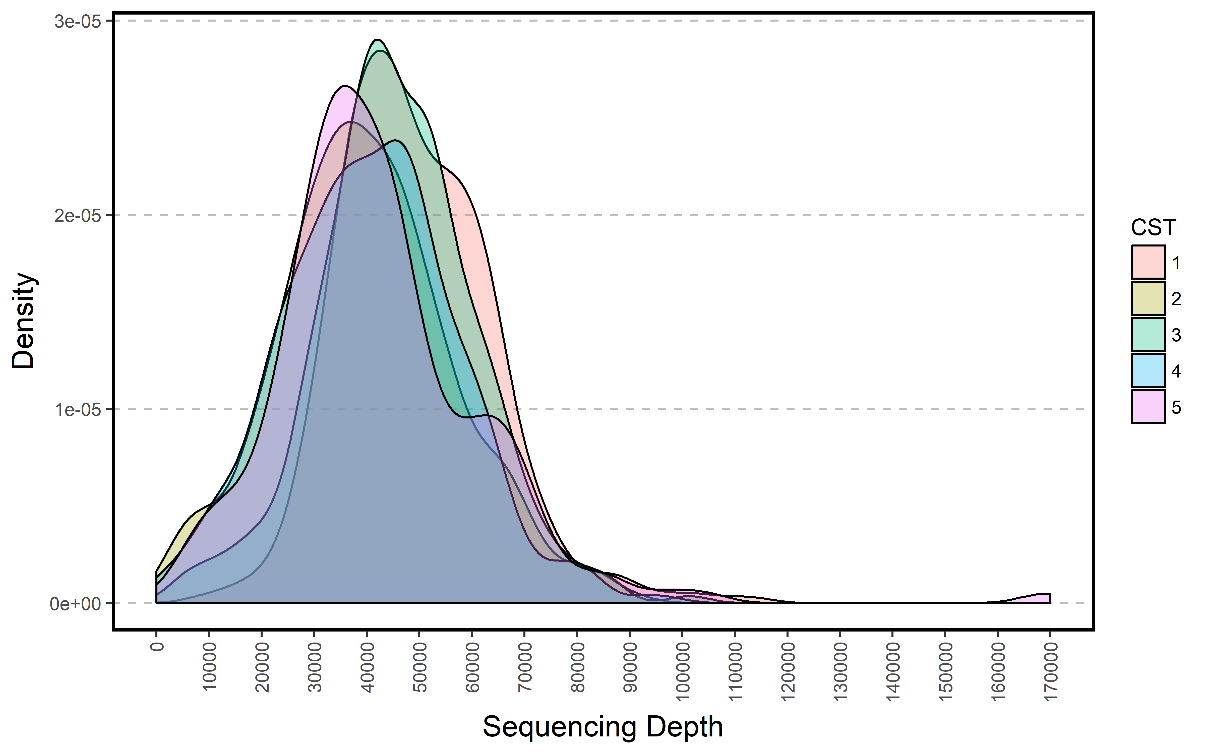


**Influenza susceptibility model, additionally adjusting for sequencing depth.**

|  | **Odds Ratio** | **95% Confidence Interval** |
| --- | --- | --- |
| CST 2 | 0.99 | (0.42, 2.31) |
| CST 3 | 0.95 | (0.41, 2.24) |
| CST 4 | 0.24 | (0.06, 0.94) |
| CST 5 | 0.38 | (0.11, 1.31) |
| 0-5 years | 4.95 | (1.71, 14.30) |
| 6-17 years | 2.97 | (1.49, 5.91) |
| Smoker in household | 0.83 | (0.40, 1.73) |
| Household crowding | 1.40 | (0.64, 3.03) |
| Sequencing depth* | 0.99 | (0.97, 1.01) |

*Units of 1,000 reads

**CST stability model, additionally adjusting for time between samples.**

|  | **Odds Ratio** | **95% Confidence Interval** |
| --- | --- | --- |
| Influenza infection | 0.78 | (0.43, 1.40) |
| CST 2 | 1.08 | (0.63, 1.87) |
| CST 3 | 1.56 | (0.90, 2.70) |
| CST 4 | 0.78 | (0.42, 1.46) |
| CST 5 | 1.26 | (0.61, 2.61) |
| 0-5 years | 0.76 | (0.37, 1.55) |
| 6-17 years | 1.67 | (1.07, 2.60) |
| Smoker in household | 0.87 | (0.59, 1.27) |
| Household crowding | 0.97 | (0.64, 1.47) |
| Days between samples | 1.01 | (0.88, 1.15) |

**Influenza susceptibility model, using 90% posterior probability criterion.**


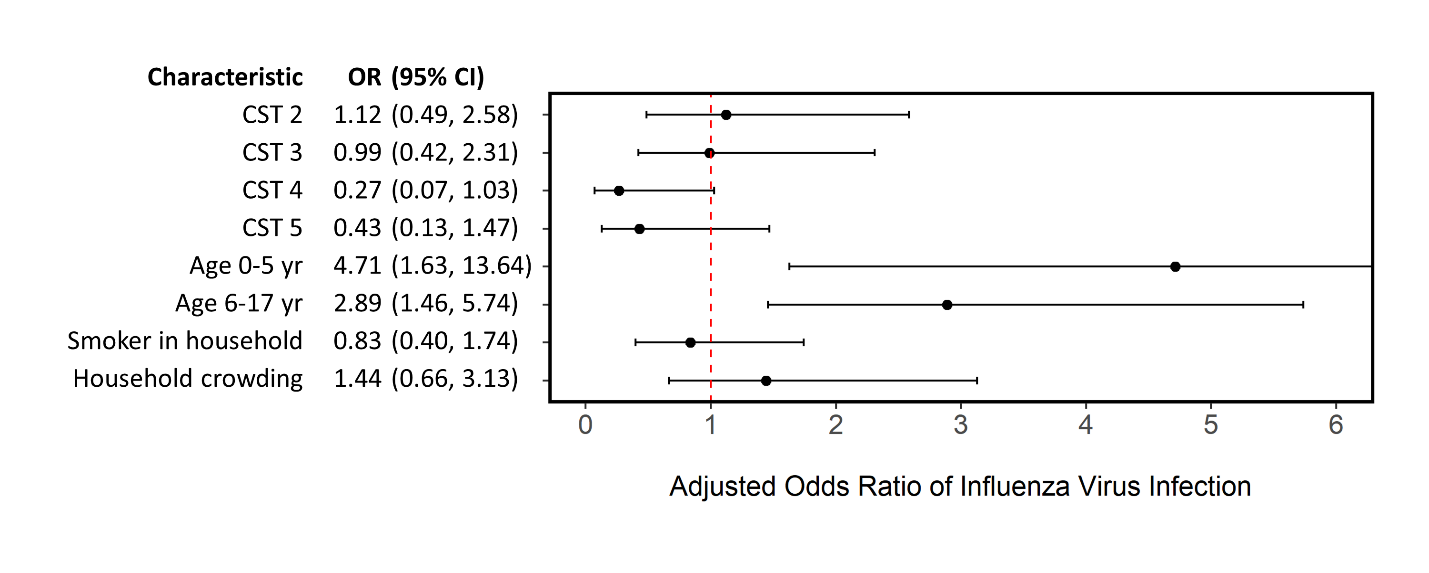


**CST stability model, using 90% posterior probability criterion.**


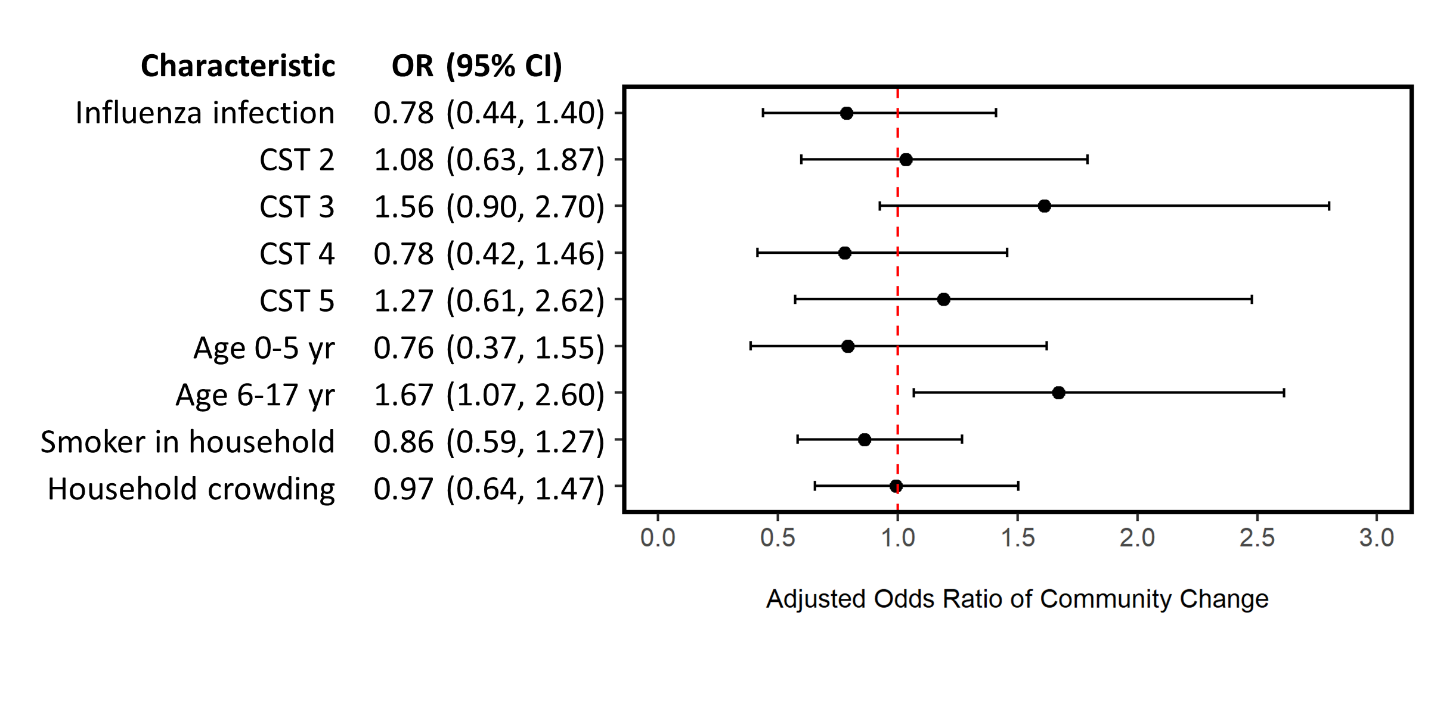


**Relationship between CST and HAI titer**

Model: $logit(Pr(Y_{ij}=1))= \beta_{0}+\beta_{1}{CST}_{ij}+\beta_{2}{Age}_{ij}+b_{0j}+\varepsilon_{ij}$

where *i* denotes person

*j* denotes household

Description:

|  | **Description** | **Variable Format** | **Type** |
| --- | --- | --- | --- |
| *Y* | ≥1:40 titer \| ≥1:20 titer | Binary factor | Outcome |
| *CST* | CST at time of enrollment | 5-level categorical factor | Fixed |
| *Age* | Age groups: 0-5 years, 6-17 years, 18-87 years | 3-level categorical factor | Fixed |
| *b_0_* | Random effects for household | Household ID as factor | Random |
| *ε* | Random error |  | Random |

H1N1 1:40 Titer

|  | Odds Ratio | 95% Confidence Interval |
| --- | --- | --- |
| CST 2 | 1.13 | (0.64, 2.00) |
| CST 3 | 0.77 | (0.44, 1.34) |
| CST 4 | 0.59 | (0.32, 1.10) |
| CST 5 | 0.88 | (0.40, 1.93) |
| Age 0-5 yr | 0.63 | (0.30, 1.32) |
| Age 6-17 yr | 2.17 | (1.39, 3.39) |

H1N1 1:20 Titer

|  | Odds Ratio | 95% Confidence Interval |
| --- | --- | --- |
| CST 2 | 1.04 | (0.57, 1.90) |
| CST 3 | 0.86 | (0.48, 1.56) |
| CST 4 | 0.65 | (0.34, 1.24) |
| CST 5 | 0.95 | (0.41, 2.17) |
| Age 0-5 yr | 0.36 | (0.17, 0.76) |
| Age 6-17 yr | 1.79 | (1.10, 2.91) |

H3N2 1:40 Titer

|  | Odds Ratio | 95% Confidence Interval |
| --- | --- | --- |
| CST 2 | 1.09 | (0.57, 2.07) |
| CST 3 | 0.85 | (0.45, 1.60) |
| CST 4 | 0.90 | (0.45, 1.80) |
| CST 5 | 0.66 | (0.28, 1.55) |
| Age 0-5 yr | 0.32 | (0.15, 0.69) |
| Age 6-17 yr | 3.34 | (1.88, 5.92) |

H3N2 1:20 Titer

|  | Odds Ratio | 95% Confidence Interval |
| --- | --- | --- |
| CST 2 | 1.03 | (0.46, 2.30) |
| CST 3 | 0.58 | (0.27, 1.24) |
| CST 4 | 0.84 | (0.35, 1.99) |
| CST 5 | 0.43 | (0.16, 1.12) |
| Age 0-5 yr | 0.32 | (0.14, 0.72) |
| Age 6-17 yr | 3.41 | (1.62, 7.18) |

B Yamagata 1:40 Titer

|  | Odds Ratio | 95% Confidence Interval |
| --- | --- | --- |
| CST 2 | 1.37 | (0.56, 3.37) |
| CST 3 | 0.98 | (0.42, 2.32) |
| CST 4 | 1.26 | (0.50, 3.17) |
| CST 5 | 1.72 | (0.51, 5.85) |
| Age 0-5 yr | 0.33 | (0.10, 1.15) |
| Age 6-17 yr | 0.86 | (0.45, 1.65) |

B Yamagata 1:20 Titer

|  | Odds Ratio | 95% Confidence Interval |
| --- | --- | --- |
| CST 2 | 1.01 | (0.46, 2.23) |
| CST 3 | 1.34 | (0.62, 2.90) |
| CST 4 | 2.22 | (0.93, 5.31) |
| CST 5 | 1.42 | (0.48, 4.26) |
| Age 0-5 yr | 0.33 | (0.11, 0.94) |
| Age 6-17 yr | 1.13 | (0.62, 2.06) |

B Victoria 1:40 Titer

|  | Odds Ratio | 95% Confidence Interval |
| --- | --- | --- |
| CST 2 | 1.57 | (0.63, 3.91) |
| CST 3 | 1.62 | (0.69, 3.80) |
| CST 4 | 0.97 | (0.38, 2.48) |
| CST 5 | 1.43 | (0.42, 4.91) |
| Age 0-5 yr | 1.29 | (0.39, 4.20) |
| Age 6-17 yr | 1.36 | (0.71, 2.61) |

B Victoria 1:20 Titer

|  | Odds Ratio | 95% Confidence Interval |
| --- | --- | --- |
| CST 2 | 1.09 | (0.51, 2.30) |
| CST 3 | 1.48 | (0.71, 3.08) |
| CST 4 | 0.87 | (0.39, 1.95) |
| CST 5 | 0.87 | (0.31, 2.42) |
| Age 0-5 yr | 1.53 | (0.57, 4.09) |
| Age 6-17 yr | 1.72 | (0.98, 3.03) |
